# Single-phase silver nanoparticles from white tea: colloidal quality, mass-specific potency and bacterial envelope permeabilisation

**DOI:** 10.1039/d6ra08462a

**Published:** 2026-09-28

**Authors:** Mohammed O. H. Jaber, Mehmet Yılmaz, Aslı Yılmaz

**Affiliations:** a Department of Molecular Biology and Genetics, Atatürk University 25240 Erzurum Türkiye mohammedoh.jaber20@ogr.atauni.edu.tr asli.yilmaz@atauni.edu.tr; b Department of Medical Sciences, University College of Science and Technology (UCST) Gizan Al-Najjar, P. O. Box 8 Khan Younis Gaza Strip Palestine; c Department of Chemical Engineering, Atatürk University 25240 Erzurum Türkiye mehmetylmz@atauni.edu.tr; d Department of Nanoscience and Nanoengineering, Atatürk University 25240 Erzurum Türkiye; e Institute for Drugs, Vaccines and Biotechnology, Atatürk University 25240 Erzurum Türkiye

## Abstract

Silver nanoparticles (AgNPs) are broad-spectrum antibacterial materials that remain active against clinically relevant pathogens, but tea-mediated syntheses have focused predominantly on green tea and have generally emphasised potency without linking particle properties to downstream damage of the bacterial envelope. Here, we synthesised AgNPs using white tea (*Camellia sinensis*) and benchmarked them against a citrate-reduced preparation produced and assayed in parallel. X-ray diffraction identified face-centred-cubic Ag in both preparations; the white-tea product showed no crystalline silver-salt impurity, while the chemical benchmark contained AgCl. The biogenic particles were smaller and less polydisperse, based on electron microscopy and light scattering, and carried a more negative zeta potential (28.3 ± 7.7 nm; *ζ* = −19.6 ± 0.8 mV; polydispersity index 0.319 *versus* 0.466). Minimum inhibitory concentrations (MICs), determined independently for *Escherichia coli* ATCC 25922 and *Staphylococcus aureus* ATCC 29213 by broth microdilution, were approximately eight-fold lower for the biogenic preparation once normalised to total silver (4.7 *versus* 38.5 µg mL^−1^). At sub-inhibitory exposures, complementary flow-cytometric, electron-microscopic and time-resolved supernatant absorbance measurements were consistent with damage to the bacterial envelope and release of intracellular material in both organisms. An *A*_320_ particle-scatter tracer additionally identified a sample-processing-related over-subtraction artefact in the *A*_260_ leakage assay. Release was detectable within 1 h, with modest subsequent increases, consistent with early envelope disruption.

## Abstract 2

White-tea silver nanoparticles are single-phase, uniformly dispersed and eightfold more potent per unit of silver than a citrate benchmark, permeabilising the envelope of both Gram-negative and Gram-positive bacteria.

## Graphical abstract

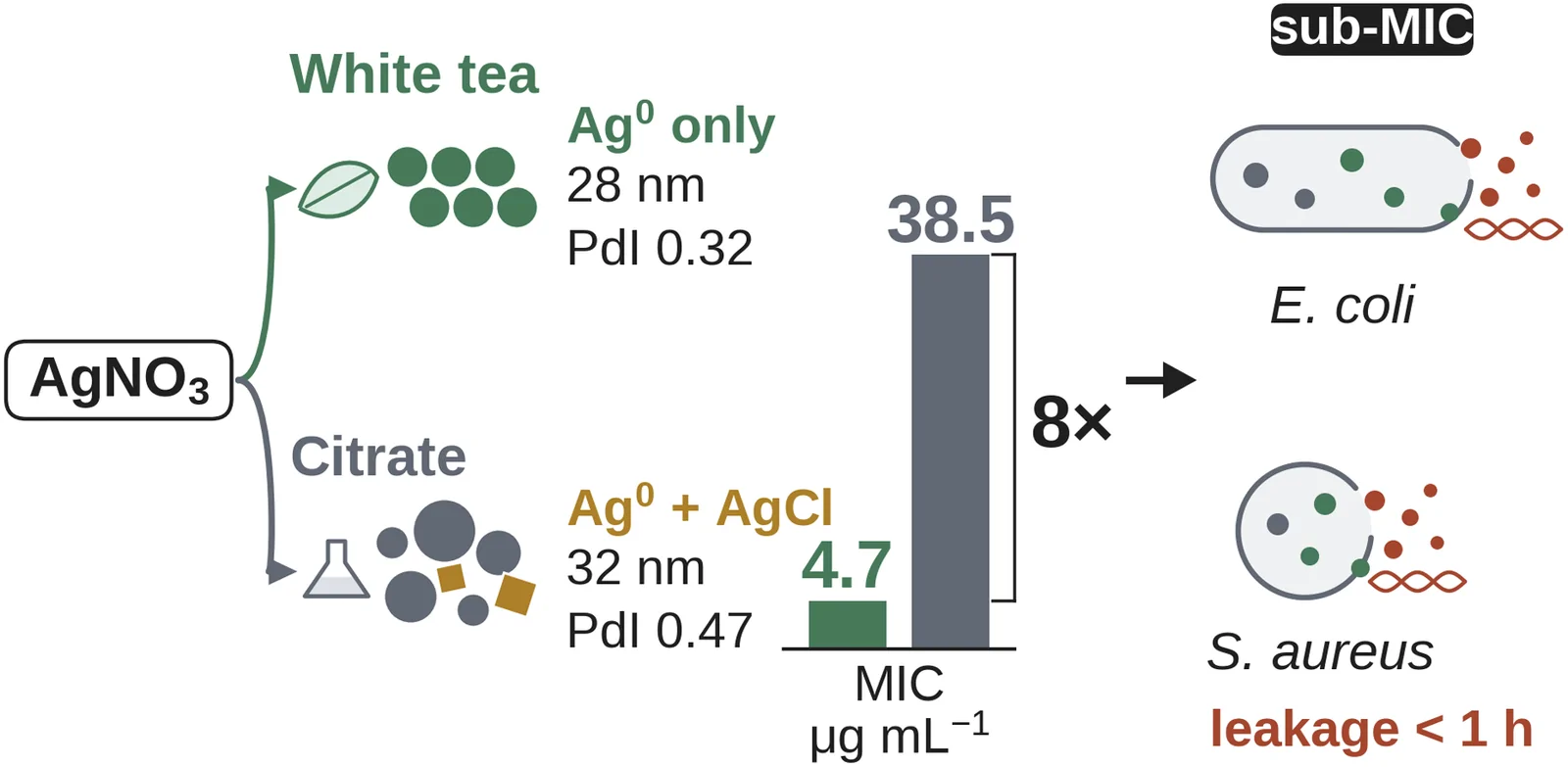

## Introduction

Bacterial infection and the accelerating spread of antimicrobial resistance (AMR) are among the most pressing challenges in contemporary medicine. The most recent Global Burden of Disease analysis estimated that in 2021, 4.71 million deaths (95% uncertainty interval 4.23–5.19 million) were associated with bacterial AMR, of which 1.14 million (1.00–1.28 million) were directly attributable to it; further, the analysis forecasted 1.91 million attributable and 8.22 million associated deaths annually by 2050.^1^ This analysis also shows the burden shifting rather than simply growing: between 1990 and 2021, AMR mortality decreased by more than half among children younger than five years but rose by over 80% among adults aged 70 years and older; deaths associated with methicillin-resistant *Staphylococcus aureus* rose from 261 000 to 550 000, and deaths associated with carbapenem resistance among Gram-negative organisms rose from 619 000 to 1.03 million.^1^ These estimates supersede the earlier figures of 1.27 million attributable and 4.95 million associated deaths reported for 2019,^2^ and they establish the urgency of the problem from observed rather than modelled data; the widely cited projection of ten million annual deaths by 2050 derives from a modelled worst-case scenario and has been critiqued on methodological grounds.^3^ Resistance arises through efflux-pump overexpression, reduced membrane permeability, target-site modification and enzymatic drug degradation, and these mechanisms progressively erode the efficacy of conventional antibiotics against both Gram-positive and Gram-negative pathogens.^4,5^

Against this backdrop, silver nanoparticles (AgNPs) have re-emerged as broad-spectrum agents that remain active against multidrug-resistant strains of *Escherichia coli* and *Staphylococcus aureus*.^6–8^ Their appeal is mechanistic rather than merely empirical. Unlike antibiotics, which typically act on a single molecular target, AgNPs attack bacteria through several simultaneous routes: sustained release of Ag^+^ ions, generation of reactive oxygen species, physical disruption of the cell envelope, and direct interaction with nucleic acids and thiol-bearing proteins. This multi-target mode of action substantially reduces the probability that a single mutational event will confer resistance,^4,5,9–11^ and it is the principal reason for silver retaining its activity where β-lactams and glycopeptides fail.^7,12,13^

The nanoscale dimensions of AgNPs confer a high surface-area-to-volume ratio and abundant reactive surface sites, and their biological performance is tunable through particle size, shape, surface charge, crystallinity and capping chemistry.^6,14–16^ Smaller particles with narrow size distributions release Ag^+^ more uniformly and penetrate the envelope more readily; more negative zeta potentials improve colloidal stability and shelf life; and the identity of the capping layer governs both dispersion behaviour and any additional intrinsic bioactivity.^17–20^

Conventional chemical and physical syntheses achieve fine control over these parameters but at a cost. Citrate (Turkevich–Frens) and borohydride (Creighton) reduction, aniline/cetyltrimethylammonium bromide systems, ascorbic-acid routes and albumin-mediated reduction all depend on reagents that are either toxic, energy-intensive or waste-generating, and several produce hazardous by-products.^16,21–24^ Reaction parameters must be tightly controlled, since minor changes in reductant concentration, pH or temperature shift the resulting size distribution and antibacterial potency.^25,26^ Comparative surveys of biological and non-biological synthesis routes consistently identify reagent toxicity and downstream waste as the decisive drawbacks of the chemical approach.^27^ A further, less frequently discussed problem is phase purity: chloride present in reagents or buffers readily precipitates as crystalline silver chloride, so that a nominally metallic preparation is in fact a two-phase Ag + AgCl system whose behaviour is not that of the intended material.

Green synthesis offers an alternative by using plant-derived polyphenols, flavonoids, catechins, reducing sugars and proteins as combined reducing and stabilising agents.^28–30^ These biomolecules can promote Ag^+^ reduction and cap the nascent particles in aqueous media while avoiding conventional toxic reductants and organic solvents.^28,30–32^ The resulting corona can also influence nucleation, growth, surface charge and biological interactions.^33–40^ Plant-mediated synthesis can therefore alter not only the reagent profile of AgNP production but also the physicochemical properties that govern antibacterial performance.

A wide range of biological feedstocks has been explored for AgNP synthesis, including higher plants, algae, fungi, yeasts, bacteria and biosurfactants.^12,32,34,35,37,40–58^ Among plant sources, tea (*C. sinensis*) is particularly attractive because its polyphenol chemistry is well characterised and its extracts contain multiple reducing and capping constituents.

Despite extensive work on tea-mediated AgNP synthesis, most reports concern green or black tea.^59–65^ White tea remains comparatively underexplored, and the three studies located that use white-tea leaf or extract each leave the present question open in a different way. Karakaş *et al.* synthesised AgNPs from white-tea leaf and did determine minimum inhibitory concentrations (MICs), in the range 0.625–10 µg mL^−1^, but against *Acinetobacter baumannii*, *Pseudomonas aeruginosa*, *Bacillus subtilis*, *Staphylococcus haemolyticus* and *Candida albicans* rather than against *E. coli* or *S. aureus*, and reported aggregated particles of 49–89 nm by scanning electron microscopy.^66^ Lakshmanan *et al.* applied white-tea-mediated AgNPs as an endodontic irrigant against *Enterococcus faecalis* and reported spherical particles of 30–35 nm by transmission electron microscopy, but expressed activity as inhibition-zone diameter at colloid volumes of 25–100 µL, so that neither an MIC nor a mass concentration can be recovered.^67^ Haghparasti and Mahdavi Shahri prepared white-tea AgNPs and a derived organo-clay nanocomposite and characterised them in detail (19.77 ± 3.82 nm by transmission electron microscopy), but evaluated only antioxidant and cytotoxic activity and carried out no bacterial testing.^68^ To our knowledge, therefore, no white-tea-mediated AgNP has been reported with an MIC determined against *E. coli* or *S. aureus*, none has been dosed against an independently measured silver content, and none has been benchmarked against a chemically prepared comparator produced and assayed in parallel. The physicochemical and antibacterial performance of white-tea-derived AgNPs is correspondingly ill-defined.

White tea is the least processed major grade of *C. sinensis*, but this does not imply uniformly higher catechin content than green tea. Comparative analyses show substantial variation among products, with green tea often containing equal or higher total catechins and polyphenols, while white tea may differ in the relative abundance of individual monomeric catechins.^66,69–71^ White tea is therefore of interest as a distinct phytochemical reducing and capping environment rather than simply as a richer catechin source. Such compositional differences could influence nucleation, corona formation and particle properties, although this possibility has not been tested directly here.

A second gap concerns mechanism. AgNP antibacterial studies frequently report inhibition zones or MIC values while inferring downstream membrane damage.^5,72,73^ Leakage measurements, when included, are often single-endpoint assays and can be confounded by nanoparticle-associated optical signals. We therefore examined two model organisms with contrasting envelope architectures—*E. coli* and *S. aureus*—and combined single-cell, ultrastructural and extracellular absorbance readouts to evaluate AgNP-associated envelope damage and intracellular-material release.^4,7,72,74^

*E. coli* ATCC 25922 and *S. aureus* ATCC 29213 are antibiotic-susceptible reference strains. They were selected for their standardised susceptibility profiles, their contrasting envelope architecture and their status as the organisms against which reference broth-microdilution procedures are calibrated. The resistance burden outlined above motivates the search for antibacterial strategies that do not depend on a single molecular target; it does not imply that the preparations characterised here were evaluated against multidrug-resistant isolates, and the present data do not speak to that question.

Accordingly, this study addressed two questions: whether white-tea-mediated synthesis produces physicochemically distinct AgNPs relative to a chemically prepared benchmark, and whether these differences are accompanied by measurable bacterial-envelope damage. Gr-AgNP and Chem-AgNP preparations were produced and assayed in parallel, characterised for crystal structure, morphology, colloidal behaviour and surface chemistry, and evaluated by MIC testing, Hoechst 33342 flow cytometry, field-emission scanning electron microscopy (SEM) and time-resolved supernatant *A*_260_ measurements. An *A*_320_ particle-scatter check was additionally used to assess residual-particle interference in the leakage assay.

## Experimental

### Nanoparticle synthesis

Two AgNP preparations were produced in parallel. The green (biogenic) route used an aqueous white-tea (*C. sinensis*) extract, whose polyphenols, catechins and soluble proteins act simultaneously as reducing and capping agents (Gr-AgNP), following the general principles established for plant-mediated synthesis.^28,30,34^ A conventional chemical reduction route (citrate reduction, targeting particles below 20 nm) produced the second preparation (Chem-AgNP).^17,21,22^

The complete synthesis protocol for both routes is reported in Section S1 and Table S8 of the SI, so that either preparation can be reproduced without recourse to secondary sources. Section S1 specifies, for the biogenic route, the white-tea mass-to-volume ratio, the extraction temperature and duration, the filtration procedure, the AgNO_3_ concentration, the extract-to-AgNO_3_ volume ratio, the reaction pH, temperature and duration, the stirring conditions and the criterion used to judge completion of the reaction; the corresponding citrate-reduction parameters for the chemical route are given in the same section. Purification and storage conditions for both preparations are also specified there.

Before physicochemical measurement, both suspensions were purified by three centrifugation cycles (12 000 rpm, 30 min each) with redispersion in distilled water between cycles, to remove unreacted precursor and loosely associated organics. Purified stocks were resuspended in ultrapure water and stored protected from light as described in Section S1, and were redispersed immediately before use. The total silver content of each purified stock was quantified by inductively coupled plasma-mass spectrometry (ICP-MS), giving approximately 102.5 µg mL^−1^ for Chem-AgNP and 28.5 µg mL^−1^ for Gr-AgNP.

### Structural and colloidal characterisation

X-ray diffraction (XRD) patterns were collected on a PANalytical EMPYREAN diffractometer (Cu Kα_1_, *λ* = 1.5406 Å; 45 kV, 40 mA) in grazing-incidence, parallel-beam geometry over 2*θ* = 10.05–89.97° with a 0.06° step. Diffractograms were background-subtracted, smoothed and phase-identified against the ICDD PDF/ICSD and COD databases using HighScore Plus; overlapping reflections were deconvoluted by Gaussian multi-peak fitting. Crystallite size (*D*) was obtained from the Scherrer equation (*D* = *Kλ*/*β* cos *θ*, *K* = 0.90), dislocation density as *δ* = 1/*D*^2^, and the cubic lattice parameter as *a* = *d*√(*h*^3^ + *k*^2^ + *l*^2^). Line broadening of the metallic-Ag reflections was further analysed *via* three Williamson–Hall (W–H) models—the uniform deformation model (UDM), the uniform stress deformation model (USDM) and the uniform deformation energy density model (UDEDM)—to separate size from strain contributions.

For SEM, particle length and projected area were measured for a representative population and fitted with a normal distribution to obtain the mean (*µ*) and standard deviation (*σ*). The chemical sample was additionally resolved by morphology (spherical *versus* rod) because visual inspection indicated a mixed population.

Hydrodynamic size (*Z*-average), polydispersity index (PdI), zeta potential and conductivity were measured on a Malvern Zetasizer-type analyser (173° backscatter detection, 25 °C, Smoluchowski approximation) in triplicate (*n* = 3). Zeta potentials were interpreted against conventional Derjaguin–Landau–Verwey–Overbeek (DLVO) stability zones. Fourier-transform infrared (FTIR) spectra of the raw white-tea extract and of Gr-AgNP were recorded over 4000–400 cm^−1^ to identify the biomolecules responsible for bioreduction and capping, and to track diagnostic band shifts occurring during synthesis.

### Bacterial strains and growth conditions

Gram-negative *E. coli* (ATCC 25922) and Gram-positive *S. aureus* (ATCC 29213) served as model pathogens. Strains were cultured in Luria–Bertani broth at 37 °C for 16–24 h, streaked to single colonies on Luria–Bertani agar and subcultured to the logarithmic phase. Cells were harvested by centrifugation (4000 rpm, 5 min) and washed three times in sterile isotonic saline to remove residual medium and metabolites. For each assay, suspensions were adjusted turbidimetrically to 0.5 McFarland (OD_600_ 0.08–0.11; approximately 1–2 × 10^8^ CFU mL^−1^).

### Antibacterial activity and minimum inhibitory concentration

Two distinct sets of measurements were made, and they are reported separately here because they serve different purposes: growth-inhibition and viability assays were used to evaluate and corroborate antibacterial activity, whereas the MIC was established by a separate, formal broth-microdilution determination.

For the activity assays, two-fold serial dilutions of each AgNP preparation were prepared in sterile broth across 96-well microplates. Equal volumes (200 µL) of the adjusted bacterial suspension (1 × 10^6^ CFU mL^−1^) and of each AgNP dilution were combined and incubated statically at 37 °C for 24 h. Growth was quantified as optical density at 600 nm on a microplate reader, and percentage inhibition was calculated relative to the untreated growth control. Every plate carried a growth control (broth plus bacteria), a sterility control (broth plus diluent) and a nanoparticle blank (broth plus AgNP, no bacteria), the last of these allowing the intrinsic absorbance of the particle suspension to be subtracted. All dilutions were run in triplicate, and the assay was repeated on independent occasions.

Metabolic viability was assessed in parallel by tetrazolium reduction, following the procedure applied to green-synthesised silver nanoparticles by Chavez-Granados *et al.*^62^ Diluted bacteria (100 µL, approximately 10^6^ CFU mL^−1^) were combined with 100 µL of the corresponding AgNP dilution and incubated at 37 °C for 4–6 h without shaking. MTT [3-(4,5-dimethylthiazol-2-yl)-2,5-diphenyltetrazolium bromide; 20 µL of a 5 mg mL^−1^ filter-sterilised solution in phosphate-buffered saline, PBS] was then added, and the plates were incubated in the dark at 37 °C for a further 2–4 h. Formazan was solubilised with 100 µL dimethyl sulfoxide and the absorbance was read at 570 nm, with viability expressed as a percentage of the untreated control. MTT was applied to bacterial cultures as a metabolic-viability readout only; no mammalian cell line was used, and this assay is therefore not a cytotoxicity determination.

The MIC itself was determined independently for each organism by broth microdilution following the Clinical and Laboratory Standards Institute (CLSI) reference procedure, and was taken as the lowest concentration at which no visible bacterial growth was observed. MIC values were therefore established separately for *E. coli* ATCC 25922 and for *S. aureus* ATCC 29213 for each preparation; the OD_600_ and MTT experiments described above were not used to define the MIC. The resulting values are given in Table 5, and the sub-inhibitory concentrations used in every subsequent mechanistic assay were set relative to them. Because the MIC of each preparation was established against its ICP-MS-determined total silver content, it is reported as an absolute mass concentration (Table 5), and the sub-inhibitory exposures derived from it are defined on the same absolute basis rather than as dilutions of a stock.

### Sub-inhibitory dosing

All mechanistic exposures were sub-inhibitory and are reported as absolute concentrations of total silver. Since the MIC of each preparation was itself fixed against its ICP-MS-determined silver content, the exposures derived from it are defined on the same absolute basis; expressing them as dilutions of a stock would add nothing once that silver content is known. Chem-AgNP was applied at 19.2 µg mL^−1^ to both organisms. Gr-AgNP was applied at 1.19 µg mL^−1^ to *E. coli* and at 2.37 µg mL^−1^ to *S. aureus*. Every concentration lay below the MIC of the organism concerned, so that the effects observed reflect early envelope events rather than gross culture collapse and so that sufficient viable biomass remained for downstream analysis. The two Gr-AgNP concentrations were established in preliminary dose-selection experiments: at 2.37 µg mL^−1^, Gr-AgNP suppressed *E. coli* growth to an extent that left too few viable cells for reliable mechanistic characterisation, whereas the same concentration produced a measurable antibacterial response in *S. aureus* while preserving viability. Each exposure was therefore chosen to be biologically informative for the organism concerned. One consequence is carried through the whole interpretation: *E. coli* and *S. aureus* received unequal Gr-AgNP exposures (1.19 *versus* 2.37 µg mL^−1^, one-quarter and one-half of the respective MIC), so the cytometric and leakage data are not read as a cross-species ranking of intrinsic potency. All exposures are collected with the corresponding MIC data in Table 5, and this constraint is restated among the limitations.

### Flow cytometry

Loss of DNA-associated fluorescence was quantified at the single-cell level after a 20 h exposure using the membrane-permeant, minor-groove double-stranded DNA stain Hoechst 33342, which enters cells irrespective of viability. An intact cell yields a strong Hoechst signal; a cell that has lost or degraded its DNA yields little signal and is recorded here as a Hoechst-low/negative event. Fluorescence was acquired in the PB450-A (Pacific Blue) channel, with 10 000 events recorded per sample. Events were first gated on forward and side scatter to exclude instrument noise and sub-bacterial debris, and the same scatter gate was applied to every sample within a run. The dye-negative/positive boundary was set from a no-dye (unstained) control and applied uniformly across all samples. A stained, untreated control was acquired for each organism in every run and consistently displayed the expected DNA-associated fluorescence, confirming that the staining procedure detected intracellular DNA under the conditions used. Experiments were performed three times on separate days using two independent synthesis batches (*n* = 3). Full acquisition settings, the gating strategy and the control panel are given in Section S2 of the SI.

#### Staining procedure

Following the 16–20 h sub-inhibitory exposure, approximately 10^6^ cells were collected from each experimental and control group, pelleted (10 000 × *g*, 1–2 min) to remove medium and unbound particles, and washed by gentle resuspension in 1× PBS. Hoechst 33342 was added to a final concentration of 5 µg mL^−1^ and the suspension was incubated for 30 min in the dark at room temperature; propidium iodide was then added to 5 µg mL^−1^ for a further 15 min in the dark. Aliquots of 300 µL of the stained suspension were transferred to microplate wells for acquisition. Acquisition was triggered on the Hoechst signal to exclude debris, and unstained, Hoechst-only and propidium iodide-only controls were run alongside every batch to set autofluorescence and compensation gates. The approach follows that of Dwyer *et al.* for stress-induced chromosomal condensation and membrane permeabilisation.^75^

Two interpretive constraints follow from this design and are applied throughout. First, a Hoechst-low/negative event indicates a reduction in detectable DNA-associated fluorescence; it is not, in itself, direct evidence of DNA release. The cytometric data are therefore interpreted alongside the supernatant Δ*A*_260_ and electron-microscopic measurements rather than in isolation. Second, nanoparticle-only fluorescence and scatter controls were not acquired, so a residual particle-associated contribution to the scatter-gated population cannot be formally excluded; this is recorded among the limitations.

### Field-emission scanning electron microscopy

#### Bacterial culture and treatment

*E. coli* ATCC 25922 and *S. aureus* ATCC 29213 were grown in Luria–Bertani broth to the logarithmic phase and standardised to 10^6^–10^8^ CFU mL^−1^. Three groups were prepared for each organism: an untreated control, a Gr-AgNP group and a Chem-AgNP group; the latter two were dosed at the sub-inhibitory concentrations of total silver specified in Table 5. Cultures were incubated for 16–20 h at 37 °C.

#### Harvesting and washing

Cells were pelleted at 5000 rpm for 5–10 min, the supernatant discarded, and the pellet resuspended in 1 mL sterile 1× PBS. This wash was repeated three times to remove culture medium and unbound nanoparticles, which would otherwise deposit as background clusters during drying.^76,77^

#### Chemical fixation

Washed pellets were resuspended in 2.5% (v/v) glutaraldehyde in 0.1 M phosphate buffer (pH 7.2–7.4) and fixed at 4 °C for at least 4 h, cross-linking the envelope in its treated state.^78,79^ Fixative was removed by three further PBS washes (5000–8000 rpm, 5 min each), the pellet being left to stand in PBS for 10 min before the final centrifugation so that residual intracellular fixative could diffuse out.

#### Dehydration and mounting

Fixed cells were dehydrated through a graded ethanol series (30, 50, 70, 80, 90 and 100% v/v; 10–15 min per step), the absolute-ethanol step being performed twice to exclude residual water. A 5–10 µL droplet of the final suspension was cast onto a silicon wafer that had been cleaned by sequential 15 min sonication in detergent solution, ethanol, acetone and ultrapure water and dried under compressed air, then mounted on double-sided carbon tape. Droplets were air-dried in a fume hood under gentle airflow to minimise the time over which surface tension acts on the envelope; hexamethyldisilazane drying and critical-point drying are alternatives where more fragile surface projections must be preserved.^80,81^

#### Coating and imaging

Dried stubs were sputter-coated with 5–10 nm of gold or gold–palladium to prevent charging. Specimens were imaged on a Zeiss field-emission scanning electron microscope using the in-lens secondary-electron detector at an accelerating voltage of 5.00 kV, a working distance of 5.2 mm and a nominal magnification of 22.62k×, and were examined for envelope pitting, shrinkage, corrugation, lysis and surface-associated particles.

#### Particle specimens

For imaging of the nanoparticles themselves, purified suspensions were centrifuged (12 000 rpm, 30 min), washed two to three times in ultrapure water to remove unreacted precursor and extract residues, resuspended and sonicated for 10 min to disperse agglomerates; the clusters seen on the substrate redisperse completely under this treatment, which distinguishes them from fused aggregates. A 5–10 µL droplet was cast onto a cleaned silicon wafer on an aluminium stub and air-dried; no conductive coating was applied (silver being conductive). Images were acquired at 5–15 kV and 30 000–100 000×. Particle diameters were measured for at least 100 particles per preparation in ImageJ (FIJI) with the scale set from the instrument scale bar, and the resulting distributions were fitted in OriginPro (Fig. S1).

### Supernatant *A*_260_ nucleic-acid leakage assay

Released nucleic acid was measured as the absorbance of the cell-free supernatant at 260 nm (NanoDrop), with *A*_280_ recorded for protein and *A*_320_ as a particle-scatter tracer; UV-grade optics were used throughout since ordinary plastics absorb at 260 nm.^82,83^ Each strain was grown to the logarithmic phase, harvested at approximately 5000 × *g* for 10 min and washed twice in PBS to remove broth-derived nucleic acid, then resuspended to 0.5 McFarland (OD_600_ ≈ 0.5), the same density being used across every set. Equal volumes were aliquoted into four sample sets prepared in triplicate for each organism, particle type and dose: buffer only (instrument blank); cells only (spontaneous-release background); AgNP only at the matched concentration, without cells (particle absorbance and scatter control); and cells + AgNP (treatment). Exposures used the sub-inhibitory concentrations of total silver given above. Because the cells were held in non-nutrient PBS at 37 °C, the population remained static and only AgNP-driven release was measured; samples were taken at 1, 2, 3 and 4 h in three technical replicates. At each time point, the suspension was centrifuged at ≥10 000 × *g* for 10 min at 4 °C to pellet both cells and nanoparticles, and the supernatant was passed through a 0.22 µm low-protein-binding filter, since centrifugation alone does not remove residual particles.^84^

A processing asymmetry was identified and corrected. The cell-containing sets were centrifuged (≥10 000 × *g*) and passed through a 0.22 µm filter, which removes bacteria and nanoparticles together, whereas the AgNP-only controls were read unprocessed. The *A*_320_ tracer was used to establish the true residual-particle background, and leakage was accordingly calculated as:Leakage = *A*_260_(cells + AgNP) − *A*_260_(cell-only)

Values are reported as mean ± SD (*n* = 3) in mAU (×10^−3^). The 0 h reading was excluded from all analyses because it is dominated by the mixing and handling contribution rather than by biological release; all means, standard deviations, control subtractions, ratios and trends therefore rest on the 1–4 h data only. The identity of the released material was assessed from the *A*_260_/*A*_280_ ratio of the treated supernatant, taking 1.8–2.1 as the nucleic-acid band; a signal that disappears once *A*_320_ is subtracted reflects residual particles rather than released nucleic acid. Two limitations specific to silver are noted: the particle-only control and the *A*_320_ correction are both mandatory, since AgNPs themselves absorb and scatter in the ultraviolet, and silver can adsorb released nucleic acid onto particles that are subsequently filtered away, which biases the measurement towards underestimation.^82–84^

### Kinetic and statistical analysis

Two complementary kinetic analyses were applied to the same 1–4 h dataset. First, leakage was regressed linearly against time across 1–4 h to detect any cumulative trend. Second, paired differences (Δ*A*_260_) were computed at the replicate level (*Δ* = later − earlier, matched by replicate index) both for consecutive intervals—*Δ*(2 − 1 h), *Δ*(3 − 2 h), *Δ*(4 − 3 h)—and relative to the 1 h reference—*Δ*(2 − 1 h), *Δ*(3 − 1 h), *Δ*(4 − 1 h). Each *Δ* was tested against zero by a one-sample *t*-test and compared with the corresponding cell-only *Δ* in a difference-in-differences framework.

Characterisation data are reported as mean ± SD (*n* = 3), with a two-tailed Welch's *t*-test used for green-*versus*-chemical contrasts. Cytometry data (*n* = 3) are summarised as mean ± SD with fold-change relative to the corresponding stained control. Leakage values are mean ± SD (*n* = 3); treated and cell-only groups were compared by independent-samples *t*-test, and the 1–4 h time trend was assessed by linear regression. A threshold of *p* < 0.05 was used throughout for the time-resolved analyses, with exact *p*-values reported; end-point leakage significance is reported as *p* < 0.0001.

## Results and discussion

### Phase composition and crystal structure

Green (Gr-AgNP) and chemical (Chem-AgNP) silver nanoparticle samples were crystalline, shown as raw and background-corrected traces against the ICDD reference patterns for Ag (01-087-0717, red sticks) and AgCl (00-001-1013, grey sticks). Both samples index to face-centred-cubic (FCC) metallic silver (space group *Fm*3̄*m*, no. 225) (Fig. 1). For the chemical sample, reflections at 2*θ* = 38.15°, 44.31°, 64.53°, 77.42° and 81.84° indexed to the (111), (200), (220), (311) and (222) planes of Ag (ICDD 01-087-0717); the green sample matched the same card through its dominant (111) reflection at 38.04°. In both preparations, the (111) reflection was strongly enhanced relative to the random-powder reference (*I*_(200)_/*I*_(111)_ ≈ 0.26 and ≈0.46, respectively, against a reference value near 0.40–0.46), indicating pronounced (111) preferred orientation. Lattice parameters (*a* = 4.085 ± 0.002 Å for the chemical sample; *a* ≈ 4.09 Å for the green sample) matched the reference value of 4.0862 Å, confirming phase-pure metallic Ag^0^ in both cases.

**Fig. 1:**
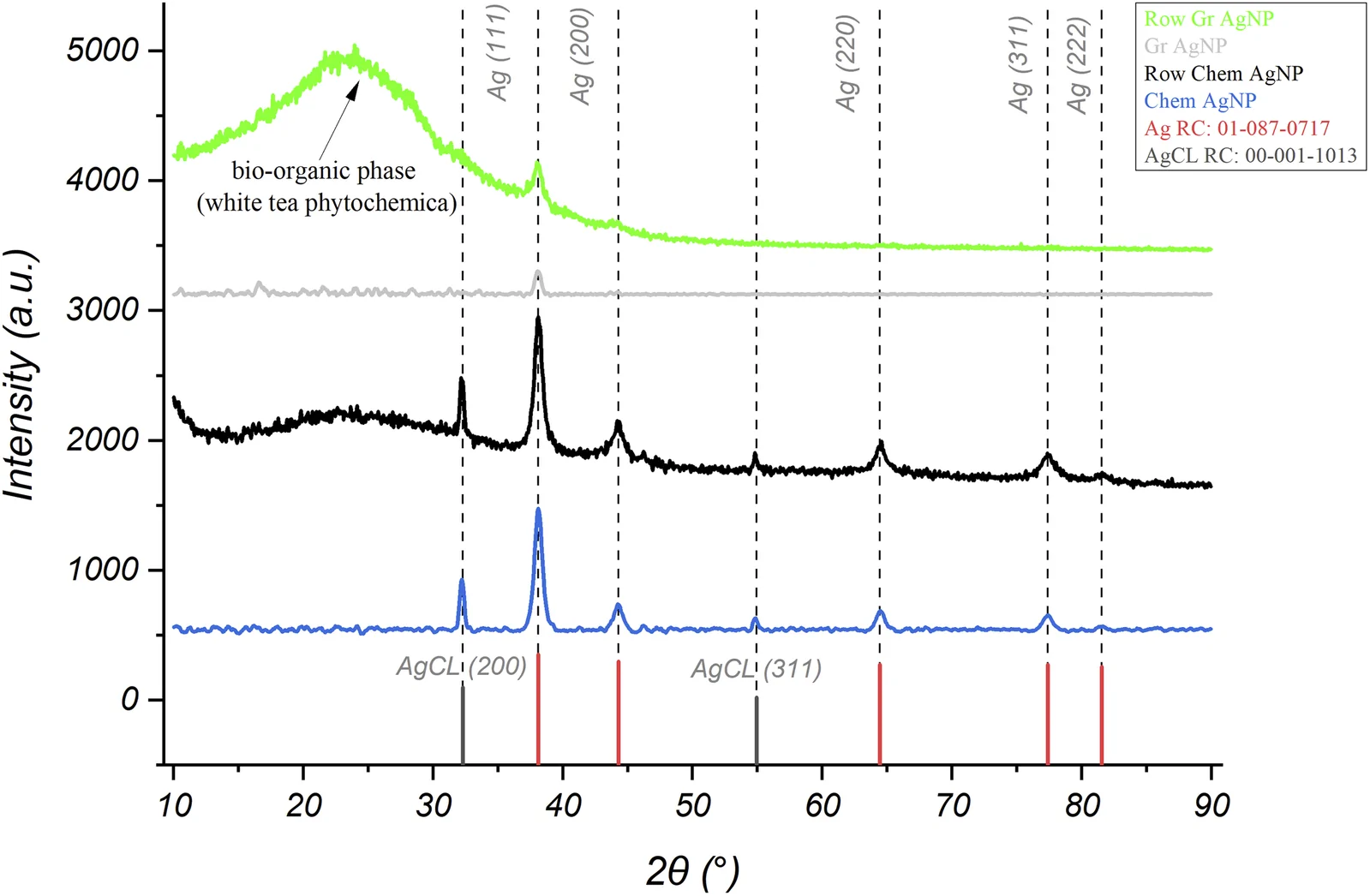
X-ray diffraction patterns of green (Gr-AgNP) and chemical (Chem-AgNP) silver nanoparticles, shown as raw and background-corrected traces against the ICDD reference patterns for Ag (01-087-0717, red sticks) and AgCl (00-001-1013, grey sticks). Both samples index to face-centred-cubic metallic silver with a dominant Ag (111) reflection at ≈38°. The chemical sample (black, raw; blue, corrected) carries additional crystalline AgCl reflections at 32.29° and 54.91°, identifying it as a two-phase Ag + AgCl product. The green sample (green, raw; grey, corrected) shows a broad, semi-crystalline bio-organic band centred near 2*θ* ≈ 23° originating from the white-tea phytochemical capping layer, with no crystalline silver-salt impurity.

The two routes differed decisively in their secondary features (Table 1). The green sample showed a broad, unindexed low-angle band (2*θ* ≈ 10–35°) attributable to a semi-crystalline bio-organic phase—the white-tea polyphenols and catechins constituting the capping corona—with no crystalline silver-salt impurity. It is therefore a single-phase metallic silver product with an amorphous organic shell. The chemical sample, by contrast, carried two additional reflections at 32.29° and 54.91°, assigned to crystalline silver chloride (chlorargyrite; ICDD 00-001-1013), making it a two-phase Ag + AgCl system with well-crystallised chloride domains. This is a synthesis contaminant rather than a designed feature, and it materially complicates interpretation of the chemical benchmark's behaviour. Phase purity of this kind is rarely reported for either green or chemical AgNP preparations, yet it determines how much of the nominal silver dose is actually available as reducible metal.

**Table 1:** Phase identification by search–match against the ICDD database. The chemical pattern is a two-phase Ag + AgCl system; for the green sample, only the Ag (111) reflection indexes to the silver card, and the remaining low-angle intensity belongs to the bio-organic phase

Sample	Phase (*hkl*)	2*θ* (°)	*d* (Å)	*I* (%)	Reference card
Chem-AgNP	AgCl (200)	32.29	2.772	31	00-001-1013
Chem-AgNP	Ag (111)	38.15	2.359	100	01-087-0717
Chem-AgNP	Ag (200)	44.31	2.044	26	01-087-0717
Chem-AgNP	AgCl (311)	54.91	1.671	10	00-001-1013
Chem-AgNP	Ag (220)	64.53	1.444	21	01-087-0717
Chem-AgNP	Ag (311)	77.42	1.233	17	01-087-0717
Chem-AgNP	Ag (222)	81.84	1.177	7	01-087-0717
Gr-AgNP	Ag (111)	38.04	2.366	100	01-087-0717

### Crystallite size and microstructure

Scherrer analysis of the (111) reflection gave a mean crystallite size of 15.6 nm for the green sample *versus* 11.1 nm for the chemical sample (Table 2). Once the sharp Ag (111) peak had been deconvoluted from the bio-organic background, the green sample's intrinsic full width at half maximum (0.537°) was appreciably narrower than that of the chemical sample (0.761°), indicating larger and better-ordered crystallites. For the chemical Ag phase, all three W–H models converged on 9.8–9.9 nm with a small compressive residual microstrain (*ε* ≈ −1.4 × 10^−3^), consistent with broadening dominated by finite crystallite size rather than by lattice strain. Dislocation density (*δ* = 1/*D*^2^) was correspondingly lower for the green crystallites (3.7 × 10^15^ m^−2^) than for the chemical Ag phase (7.0 × 10^15^ m^−2^), again reflecting the better-ordered green product. Reported sizes are uncorrected Scherrer values and are therefore lower-bound estimates.

**Table 2:** Microstructural parameters. Williamson–Hall analysis was viable only for the chemical Ag phase, which presented four indexable reflections; the green crystallite size is quoted from the Scherrer analysis. The chemical AgCl secondary phase has larger domains (*D* ≈ 21–24 nm) and lower dislocation density (*δ* ≈ 1.8 × 10^15^ m^−2^)

Parameter	Gr-AgNP (Ag)	Chem-AgNP (Ag phase)
Scherrer size, (111) (nm)	15.6	11.1
W–H UDM: size (nm)/*ε*	—	9.9/−1.4 × 10^−3^
W–H USDM: size (nm)/*σ*	—	9.8/−0.18 GPa
W–H UDEDM: size (nm)/*u*	—	9.9/0.13 kJ m^−3^
W–H fit quality, *R*^2^	—	0.64
Dislocation density *δ* (m^−2^)	3.7 × 10^15^	7.0 × 10^15^

### Particle size and morphology

Electron microscopy resolved a clear difference in the size and shape distributions produced by the two routes (Fig. 2, 3 and Table 3). Gr-AgNP formed a single population with a narrow, symmetric length distribution (*µ* = 28.3 nm, *σ* = 7.7 nm; coefficient of variation, CV ≈ 27%), and the micrographs show discrete quasi-spherical particles of uniform contrast (Fig. 2a). Chem-AgNP was markedly heterogeneous, comprising a spheroidal sub-population (≈85% by number; *µ* = 30.3 nm, CV ≈ 34%) together with longer and far more dispersed rod-like particles (≈15%; *µ* = 40.8 nm, CV ≈ 78%) that generated a pronounced right-skewed tail extending to approximately 140 nm; both habits are directly visible in Fig. 2b. Even the spheroidal sub-population of the chemical preparation was broader than the entire green population. The white-tea phytochemicals thus exerted tighter control over nucleation, growth and capping, yielding a smaller and more monodisperse product, whereas citrate reduction generated two coexisting habits. Such behaviour is consistent with that reported for biomolecule-rich reduction media, in which polyfunctional capping agents adsorb across the growing crystal faces and suppress anisotropic extension.^28,33,36^ The contrast between the two routes is therefore one of distribution—in size and in shape—rather than a uniform change in the morphology of every particle.

**Fig. 2:**
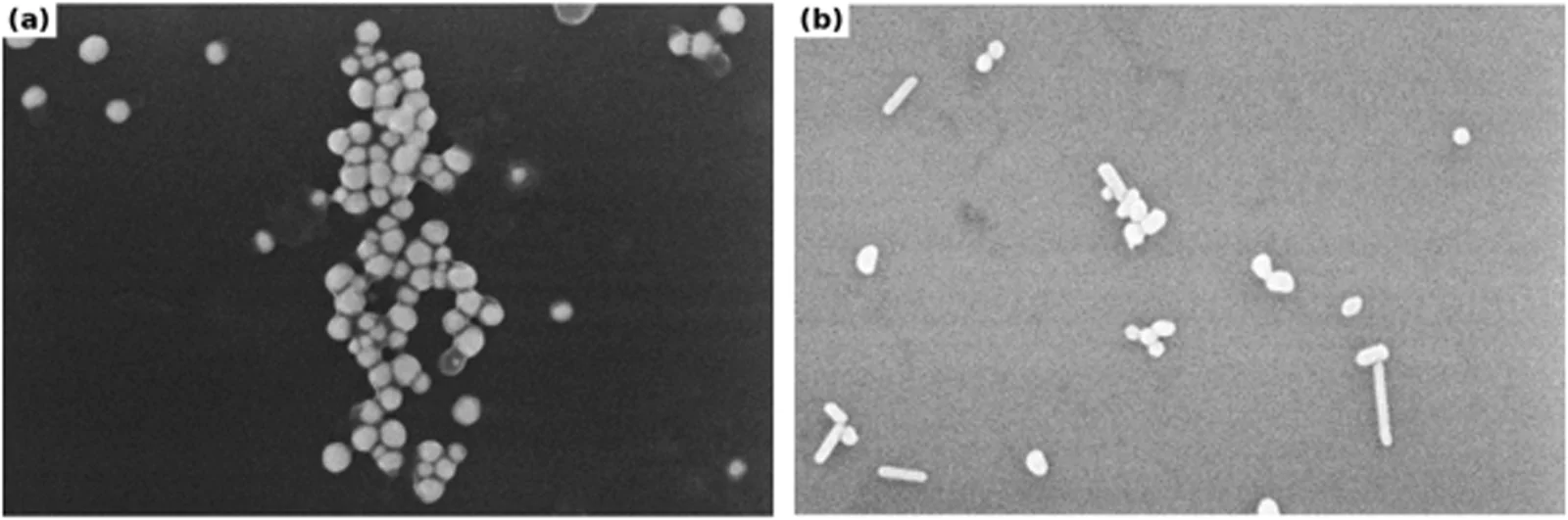
Field-emission scanning electron micrographs of the nanoparticles themselves, recorded on washed, ultrasonically dispersed suspensions drop-cast onto cleaned silicon wafers and imaged without conductive coating. (a) Gr-AgNP: a single population of discrete, quasi-spherical particles of uniform contrast, together with loose clusters in which the individual spheres remain separately resolvable and no fused necks or coalesced particle boundaries are apparent. (b) Chem-AgNP: two coexisting morphologies, comprising elongated rod-like particles alongside smaller spheroidal particles. These micrographs are the primary evidence for the distributional contrast quantified in Fig. 3 and should be read with it: the distinction between the two routes is of size and shape distribution rather than of a uniform morphological change.

**Fig. 3:**
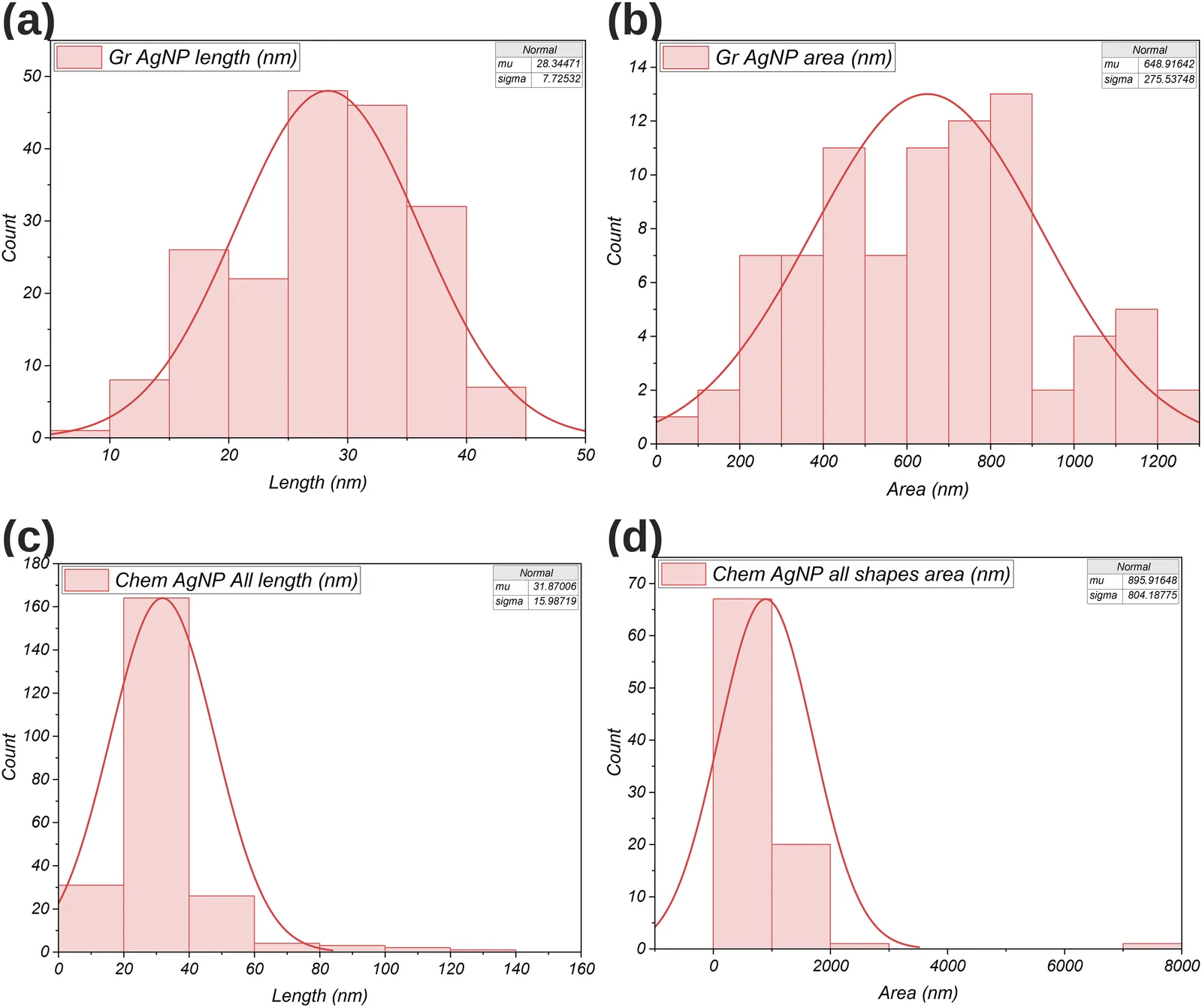
Scanning electron microscopy-derived particle-size distributions with Gaussian fits: (a and b) length and projected area of Gr-AgNP and (c and d) length and projected area of Chem-AgNP across all morphologies. The green population is narrower and single-moded (*µ* = 28.3 nm, *σ* = 7.7 nm); the chemical population is broader and right-skewed (*µ* = 31.9 nm, *σ* = 16.0 nm), with a tail reaching approximately 140 nm. The morphology-resolved distributions for the chemical sample are given in Fig. S1.

**Table 3:** SEM-derived particle-size distribution parameters (*µ* = mean, *σ* = standard deviation, CV = coefficient of variation), resolved by morphology for the chemical sample

Sample/population	Metric	*µ*	*σ*	CV (%)
Gr-AgNP (single population)	Length (nm)	28.3	7.7	27
Gr-AgNP	Area (nm^2^)	649	276	42
Chem-AgNP—all shapes	Length (nm)	31.9	16.0	50
Chem-AgNP—spherical (≈85%)	Length (nm)	30.3	10.4	34
Chem-AgNP—rod (≈15%)	Length (nm)	40.8	31.8	78

The particle associations visible in Fig. 2a are clusters rather than aggregates in the strict sense, and the distinction is worth drawing explicitly because the two terms carry different physical meanings. Following standard nanotechnology usage, an aggregate is an assembly of strongly bonded or fused primary particles whose combined external surface area is appreciably smaller than the sum of the areas of its constituents, and which is not readily redispersed; an agglomerate, or cluster, is a weakly bound assembly that essentially retains the surface area of its constituent particles and can be broken up by moderate energy input. Two observations place the present material unambiguously in the latter category. First, the individual spheres remain separately resolvable throughout each assembly, and no fused necks, sintered contacts or coalesced boundaries are visible between neighbouring particles. Second, and decisively, the assemblies are reversible: 10 min of ultrasonication during specimen preparation redisperses them into discrete particles, which would not be achievable for fused or sintered aggregates. The clustering observed by electron microscopy therefore reflects drying-induced assembly on the substrate, together with the moderate electrostatic stabilisation recorded by electrophoretic light scattering (*ζ* = −19.6 ± 0.8 mV), rather than irreversible particle fusion during synthesis. This is consistent with the hydrodynamic diameter exceeding the primary particle size while the polydispersity index remains moderate, and it distinguishes the present preparation from white-tea particles reported previously as aggregated.^66^

### Colloidal stability and surface charge

Both samples carried a net negative surface charge, but the green dispersion was significantly more negative (*ζ* = −19.6 ± 0.8 mV) than the chemical dispersion (−13.4 ± 0.7 mV; Welch's *t*-test, *p* ≈ 6 × 10^−4^), a result corroborated by the electrophoretic mobilities (−1.537 *versus* −1.050 µm cm V^−1^ s^−1^) (Fig. 4 and Table 4). On the conventional DLVO interpretation, the chemical sample sits within the incipient-instability zone (|*ζ*| = 10–20 mV), whereas the green sample lies at its upper edge and gains additional steric stabilisation from its biomolecular corona—a combination of electrostatic and steric stabilisation that is characteristic of well-capped phytosynthesised particles.^35,37,51^ Critically, the polydispersity ordering matched the SEM result: the green sample was significantly less polydisperse (PdI 0.319 *versus* 0.466; *p* ≈ 7 × 10^−3^). Two methodologically independent techniques therefore agree that the white-tea route yields the more uniform product. Intensity-weighted *Z*-average diameters exceed the primary SEM and XRD sizes, as expected for values influenced by the hydration shell and by transient clustering.

**Fig. 4:**
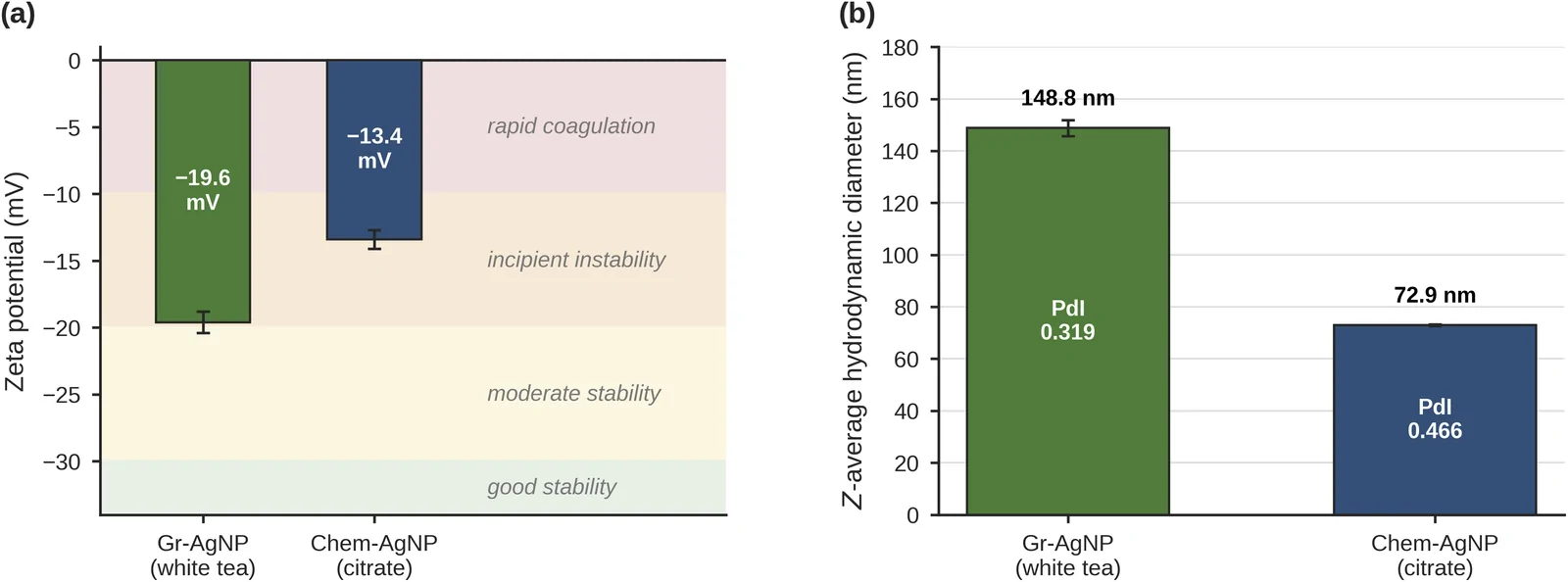
Colloidal characterisation by dynamic and electrophoretic light scattering (mean ± SD, *n* = 3). (a) Zeta potential plotted against conventional Derjaguin–Landau–Verwey–Overbeek stability zones; the green sample lies at the upper edge of the incipient-instability zone, while the chemical sample sits within it. (b) *Z*-Average hydrodynamic diameter with the polydispersity index annotated on each bar; the green sample is the less polydisperse of the two. Error bars represent ±1 SD.

**Table 4:** Colloidal properties determined from dynamic and electrophoretic light scattering (mean ± SD, *n* = 3; two-tailed Welch's *t*-test)

Parameter	Gr-AgNP	Chem-AgNP	Significance
Zeta potential (mV)	−19.6 ± 0.8	−13.4 ± 0.7	*p* ≈ 6 × 10^−4^
Electrophoretic mobility (µm cm V^−1^ s^−1^)	−1.537 ± 0.062	−1.050 ± 0.049	—
*Z*-Average diameter (nm)	148.8 ± 3.1	72.9 ± 0.3	*p* ≈ 5 × 10^−4^
Polydispersity index	0.319 ± 0.031	0.466 ± 0.013	*p* ≈ 7 × 10^−3^
Conductivity (mS cm^−1^)	0.024 ± 0.006	0.018 ± 0.000	—

### Surface chemistry: bioreduction and capping

FTIR spectroscopy confirmed that white-tea biomolecules mediate both reduction and capping (Fig. 5 and Table S1). Comparison of the raw extract with Gr-AgNP showed diagnostic shifts consistent with polyphenol and protein engagement of the silver surface: the O–H/N–H stretch shifted from 3300 to 3321 cm^−1^ (+21 cm^−1^), the protein C=O/amide I band from 1605 to 1636 cm^−1^ (+31 cm^−1^), the C–O/C–N band of alcohols, phenols and sugars from 1040 to 1001 cm^−1^ (−39 cm^−1^), and a lower-fingerprint framework/Ag–O feature from 610 to 615 cm^−1^ (+5 cm^−1^). The two largest shifts identify the carbonyl and C–O functionalities as most directly involved in Ag^+^ reduction and subsequent particle capping, in agreement with the mechanism reported across the phytosynthesis literature.^30,33,45,48^ The chemical sample showed no comparable organic band structure, consistent with a citrate-only surface.

**Fig. 5:**
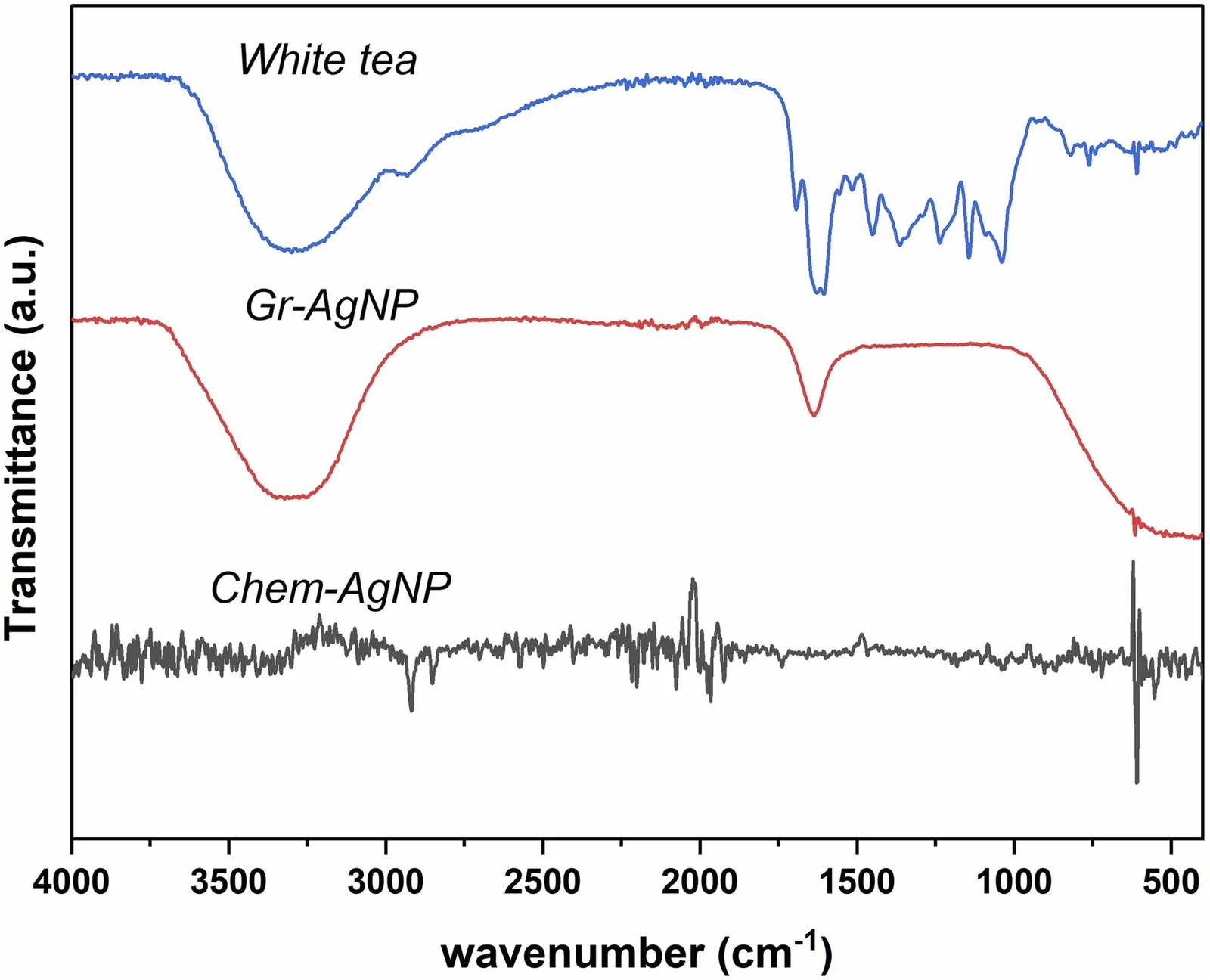
Fourier-transform infrared transmittance spectra of the raw white-tea extract (blue), Gr-AgNP (red) and Chem-AgNP (grey) over 4000–400 cm^−1^, offset vertically for clarity. The extract shows the full complement of polyphenol and protein bands; these are retained in attenuated and shifted form in Gr-AgNP, confirming that white-tea biomolecules are carried through onto the particle surface as a capping corona. The diagnostic shifts for Gr-AgNP belong to the C=O/amide I band (1605 → 1636 cm^−1^) and the C–O band (1040 → 1001 cm^−1^). The chemical sample shows no comparable organic band structure, consistent with a citrate-only surface.

### Minimum inhibitory concentration and mass-specific potency

Broth microdilution, performed independently for each organism, established the potency scale on which every subsequent mechanistic exposure was set (Table 5); OD_600_ growth inhibition and MTT viability were measured in parallel as supporting evidence of antibacterial activity. Expressed as total silver quantified by ICP-MS, the chemically reduced preparation inhibited growth at 38.5 µg mL^−1^ and the white-tea preparation at 4.7 µg mL^−1^. Expressed per unit of total silver, the green particles were therefore approximately eightfold more potent than the chemical benchmark, and they achieved this despite a stock containing less than a third as much silver (28.5 *versus* 102.5 µg mL^−1^).

**Table 5:** Total silver content, MIC and the sub-inhibitory exposures used in the mechanistic assays. Silver content was quantified by ICP-MS. MIC was determined first for each preparation by CLSI broth microdilution, independently against *E. coli* ATCC 25922 and *S. aureus* ATCC 29213; the sub-inhibitory exposures used in the mechanistic assays were then set relative to those values. All concentrations are absolute values of total silver. The final row expresses each exposure as a ratio to the corresponding organism's own MIC and shows that the two Gr-AgNP exposures are not equivalent between species

Parameter	Gr-AgNP (white tea)	Chem-AgNP (citrate)
Total silver in stock, ICP-MS (µg mL^−1^)	28.5	102.5
MIC, *E. coli* and *S. aureus* (µg mL^−1^)	4.7	38.5
Relative mass-specific potency	8.1×	1.0× (reference)
Sub-inhibitory exposure, *E. coli* (µg mL^−1^)	1.19	19.2
Sub-inhibitory exposure, *S. aureus* (µg mL^−1^)	2.37	19.2
Exposure as a ratio of the organism's own MIC	0.25 (*E. coli*); 0.50 (*S. aureus*)	0.50 (both organisms)

This ordering is consistent with the physicochemical contrasts described above. A smaller, single-mode, single-phase particle presents more surface area per unit mass for Ag^+^ release than a polydisperse population, part of which has silver locked in sparingly soluble AgCl domains, and the retained polyphenol corona may contribute additively. Two qualifications should be stated. First, because the same MIC endpoint was reached against both species for a given preparation, the mass-specific contrast between the two preparations is not confounded by species-specific differences in the MIC itself; equally, the data do not resolve differential susceptibility between the two organisms. Second, the MIC fixes the dose scale rigorously but does not by itself identify which particle property is responsible for the difference, since neither Ag^+^ release nor corona composition was quantified directly here.

### Sub-inhibitory exposure increases the Hoechst-low/negative cell fraction

In both organisms, treatment with green or chemical AgNP raised the proportion of Hoechst-low/negative events above the stained, untreated control (Fig. 6 and Table 6; replicate-level data in Table S2). The response was most marked in *E. coli*, where the Hoechst-low/negative fraction rose from 1.20% ± 0.17% in the control to 9.61% ± 0.49% with Chem-AgNP (8.0-fold) and 3.56 ± 0.44% with Gr-AgNP (3.0-fold). In *S. aureus*, which carried a substantially higher baseline (12.95% ± 0.80%), the fraction increased to 18.06% ± 1.29% with Chem-AgNP (1.4-fold) and 22.99% ± 1.28% with Gr-AgNP (1.8-fold). The increases were reproducible across three independent runs and were mirrored by a corresponding fall in the Hoechst-positive population. Notably, the ranking inverted between species: Chem-AgNP produced the larger effect in *E. coli*, whereas Gr-AgNP produced the larger effect in *S. aureus*. Because a Hoechst-low/negative event reports a loss of detectable DNA-associated fluorescence rather than DNA release *per se*, these data are treated as one of three convergent lines of evidence and are considered below together with the ultrastructural and extracellular-absorbance measurements.

**Fig. 6:**
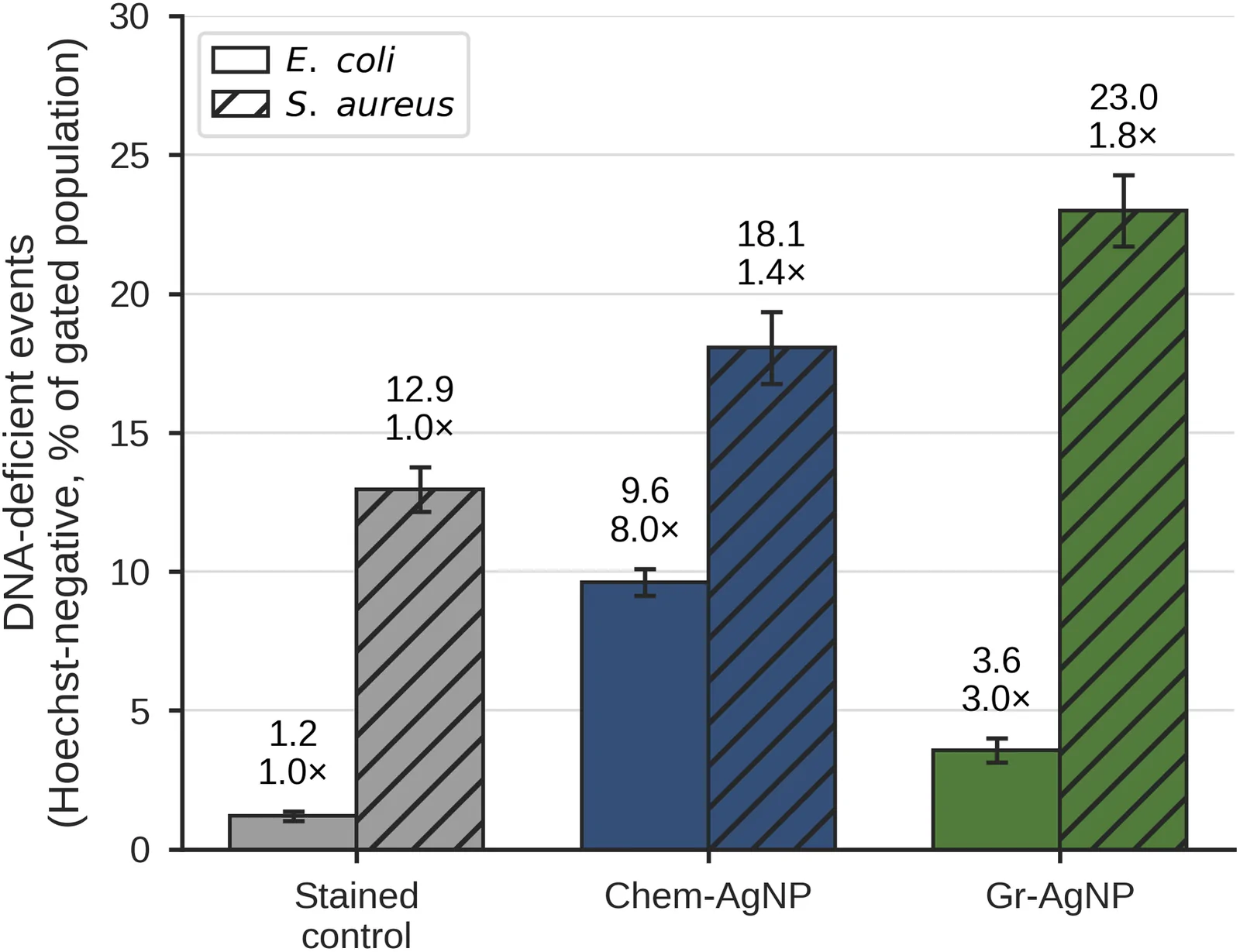
Mean Hoechst-low/negative event fraction (±SD, *n* = 3) in *Escherichia coli* (plane shading) and *Staphylococcus aureus* (hatched shading), expressed as a percentage of the scatter-gated population, with fold-change *versus* the corresponding stained control in parentheses. Both AgNP preparations raised the fraction above control in each organism, but the ranking inverts between species: Chem-AgNP gives the larger response in *E. coli* (8.0×), whereas Gr-AgNP gives the larger response in *S. aureus* (1.8×). Note the high *S. aureus* baseline (12.95%), which compresses the detectable dynamic range in that organism.

**Table 6:** Hoechst-low/negative (PB450-A) events as a percentage of the scatter-gated population (mean ± SD, *n* = 3). Exposures are absolute concentrations of total silver, as set out in Table 5. “Fold” is the mean relative to the corresponding stained control

Organism	Treatment	Hoechst-low/negative events (%)	Fold
*E. coli*	Stained control	1.20 ± 0.17	1.0×
*E. coli*	Chem-AgNP, 19.2 µg mL^−1^	9.61 ± 0.49	8.0×
*E. coli*	Gr-AgNP, 1.19 µg mL^−1^	3.56 ± 0.44	3.0×
*S. aureus*	Stained control	12.95 ± 0.80	1.0×
*S. aureus*	Chem-AgNP, 19.2 µg mL^−1^	18.06 ± 1.29	1.4×
*S. aureus*	Gr-AgNP, 2.37 µg mL^−1^	22.99 ± 1.28	1.8×

### Ultrastructural evidence: ghost and ruptured cells

To test whether the Hoechst-low/negative events correspond to cells that have physically lost their contents, AgNP-treated *E. coli* were examined by field-emission SEM (Fig. 7). Treated cells had lost the smooth, turgid rod morphology characteristic of intact *E. coli* and instead appeared as collapsed, wrinkled and deflated envelopes, frequently fused into aggregated masses bearing surface debris. These ghost-like, ruptured envelopes are the morphological counterpart of an emptied cell and provide independent ultrastructural support for the cytometric finding that AgNP exposure permeabilises the envelope and causes loss of intracellular material. Comparable envelope collapse has been documented for silver-treated *E. coli* by several groups.^18,74,85^

**Fig. 7:**
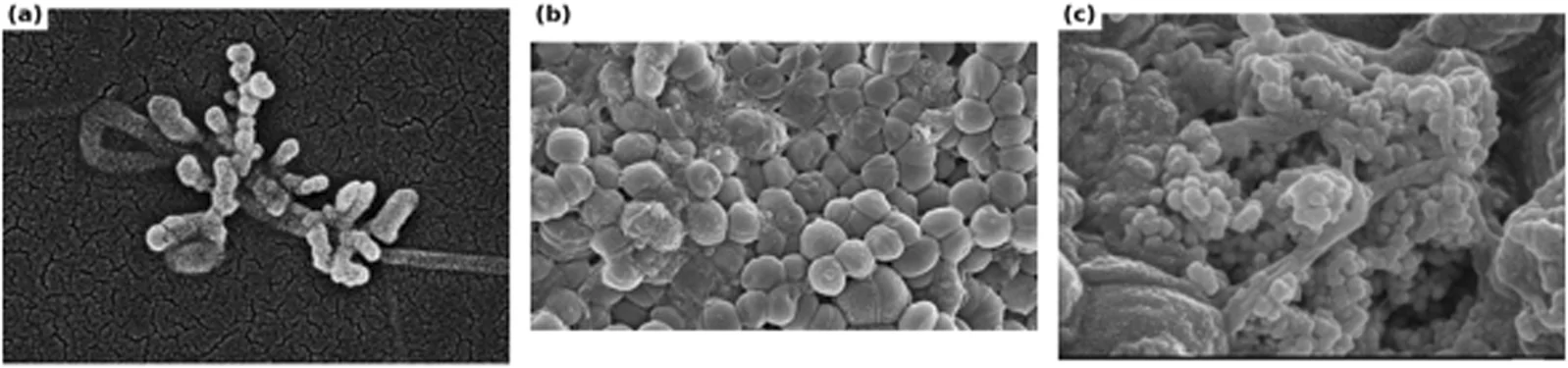
Field-emission scanning electron micrographs of bacteria after sub-inhibitory AgNP exposure. (a) *Escherichia coli* treated with Gr-AgNP: rod cells carry dense surface-associated particulate material, and two collapsed, flattened envelopes retaining the outline of a rod but no internal volume are visible in the lower left section of the field—the “ghost” morphology expected of a cell has lost its cytoplasmic contents. (b) *Staphylococcus aureus* treated with Gr-AgNP: cocci show discrete particulate clusters bound to the surface and localised surface craters, and individual cells have deflated into concave, wrinkled forms while neighbouring cells remain turgid. (c) *S. aureus* treated with Chem-AgNP: cells are largely obscured beneath a confluent particulate crust, and the cell outlines are no longer resolvable. The micrographs are qualitative and sample a small number of fields; they are presented as one of the three convergent readouts (Table S7) and not as a quantitative measure of the proportion of the population affected.

### Correction of a particle-removal artefact in the *A*_260_ leakage assay

The final and decisive step measured the released material itself. Before the biology could be interpreted, the assay had to be corrected for a sample-processing artefact relevant to the field. The cell-containing sets were centrifuged and 0.22 µm-filtered, a procedure that removes bacteria and nanoparticles together, whereas the AgNP-only control was read without that particle removal. Subtracting the unprocessed, particle-rich control therefore over-corrected the filtered treated samples and drove the green-AgNP estimates spuriously negative.

The *A*_320_ particle-scatter tracer confirmed this diagnosis unambiguously (Fig. 8d and Table S3). Treated supernatants read *A*_320_ = 0.0523–0.0540, at or below the cell-only level (0.0566–0.0578) and far below the unprocessed AgNP-only controls (0.0808 for chemical, 0.0828 for green). Expressed as a residual particle load relative to the cell-only baseline, the treated samples sat at −4% to −7%: the residual particle content of the treated supernatant, and hence its contribution at 260 nm, is effectively zero. The cell-only control is therefore the correct and only valid background. Given how frequently *A*_260_ leakage is reported in the nanomaterials literature and how rarely the particle background is verified independently, this correction may have implications well beyond the present dataset.

**Fig. 8:**
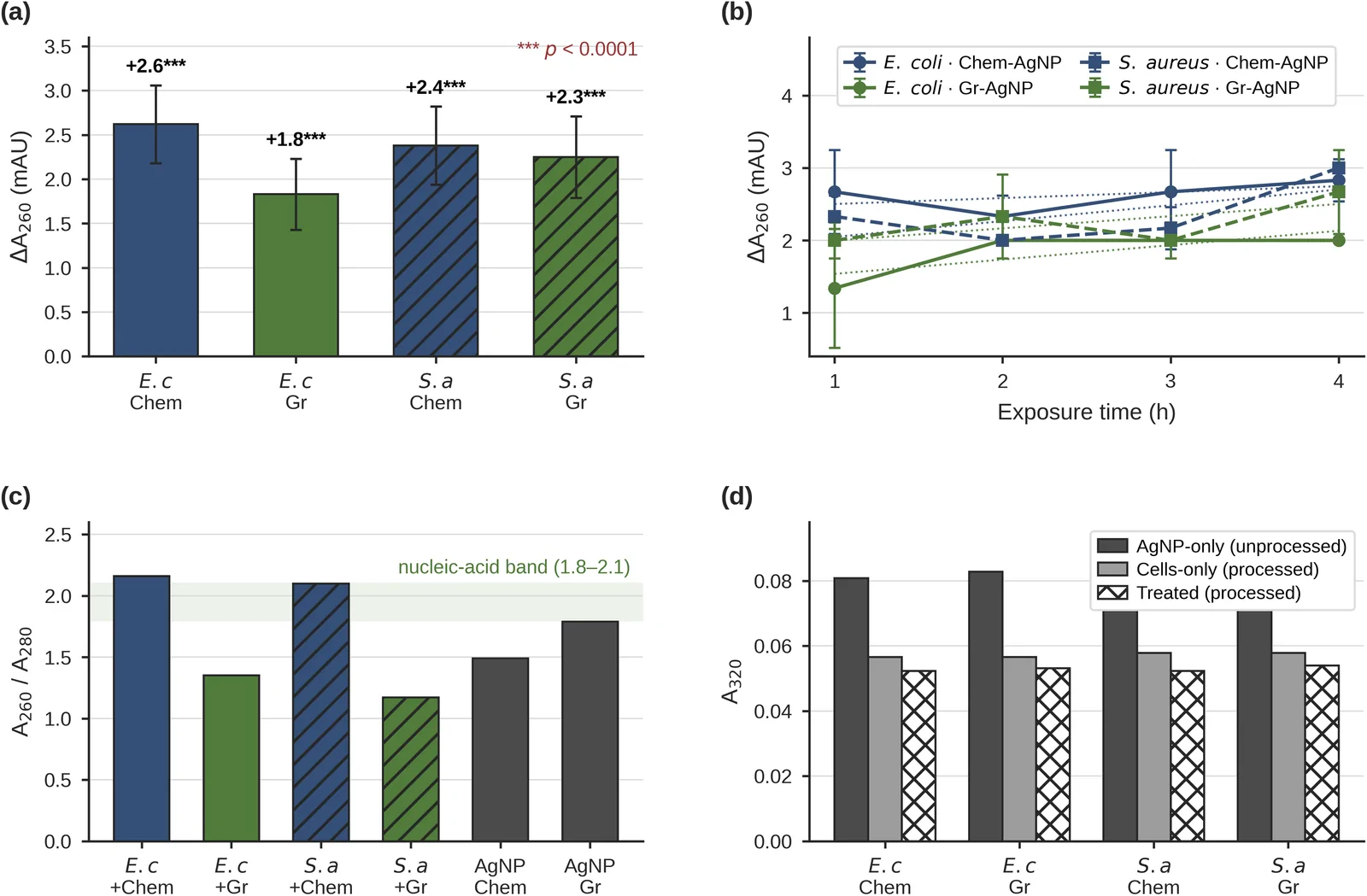
AgNP-associated extracellular *A*_260_ increase from *Escherichia coli* and *Staphylococcus aureus* over the 1–4 h exposure window (mean ± SD, *n* = 3). (a) Pooled leakage, calculated as treated minus the cell-only control; all four organism/particle combinations are significantly positive (***, *p* < 0.0001). (b) Leakage resolved by exposure time with linear trends (dotted); every group returns a positive slope, so release does not plateau within the window. (c) *A*_260_/*A*_280_ of the supernatant against the 1.8–2.1 band expected for nucleic acid; the chemical-particle supernatants fall within it, and the green-particle supernatants are below it. (d) *A*_320_ particle-scatter tracer: filtered treated supernatants sit at or below the cell-only level and far below the unprocessed AgNP-only control, supporting the use of the cell-only signal as the most appropriate background under the present processing conditions. Plain bars and markers denote *E. coli*; hatched bars and dashed lines denote *S. aureus*. Navy denotes Chem-AgNP and green denotes Gr-AgNP throughout.

### Magnitude, identity and kinetics of nucleic-acid release

With the residual-particle background correctly set, leakage (treated − cell-only) was positive and highly significant in all four organism/particle combinations, ranging from +1.8 to +2.6 mAU (all *p* < 0.0001) (Fig. 8a and Table 7). The earlier negative green-AgNP values were an artefact of over-subtraction and disappeared entirely under the corrected calculation. Both nanoparticle types therefore breach the envelope in both organisms.

**Table 7:** Nucleic-acid leakage (treated − cell-only) per timepoint and overall, in mAU (mean ± SD, *n* = 3), with the *A*_260_/*A*_280_ ratio of the treated supernatant and the linear-regression slope across 1–4 h

Organism	AgNP	1 h	2 h	3 h	4 h	Overall	*p*	*A*_260_/*A*_280_	Slope (mAU h^−1^); *p*
*E. coli*	Chem-AgNP	+2.7	+2.3	+2.7	+2.8	+2.6 ± 0.4	<0.0001	2.17	+0.08; 0.4825
*E. coli*	Gr-AgNP	+1.3	+2.0	+2.0	+2.0	+1.8 ± 0.4	<0.0001	1.35	+0.20; 0.0392
*S. aureus*	Chem-AgNP	+2.3	+2.0	+2.2	+3.0	+2.4 ± 0.4	<0.0001	2.11	+0.22; 0.0460
*S. aureus*	Gr-AgNP	+2.0	+2.3	+2.0	+2.7	+2.3 ± 0.5	<0.0001	1.17	+0.17; 0.1626

The composition of the released material was assessed using the *A*_260_/*A*_280_ ratio (Fig. 8c). Chem-AgNP-treated supernatants yielded ratios of 2.17 for *E. coli* and 2.11 for *S. aureus*, supporting a substantial nucleic-acid contribution. In contrast, Gr-AgNP-treated supernatants gave lower ratios of 1.35 and 1.17, respectively, indicating a larger relative contribution from proteins and/or corona-derived components. Extracellular UV-absorbing material was therefore clearly detected after Gr-AgNP treatment, but its identification as predominantly nucleic acid is less certain. Direct DNA-specific quantification would be required to resolve this point conclusively.

Two complementary kinetic analyses were applied to the same 1–4 h dataset, and together they give a more precise picture than either alone. Linear regression across the window shows that leakage does not plateau (Fig. 8b and Table 7). Every group returned a positive slope, ranging from +0.08 to +0.22 mAU h^−1^, and the increase reached statistical significance in two of the four combinations: *E. coli* + Gr-AgNP (+0.20 mAU h^−1^, *p* = 0.0392) and *S. aureus* + Chem-AgNP (+0.22 mAU h^−1^, *p* = 0.0460).

The paired between-timepoint (delta) analysis explains why this trend was not previously detected. Throughout the window, the treated supernatants sat at approximately 2–3 mAU, while the cell-only controls remained at or near 0 mAU at every timepoint (Fig. 9a and Table S4). Individual step-to-step changes, whether consecutive or referenced to 1 h, were small (magnitude ≤ ≈0.7 mAU) and scattered around zero, with no consistent direction (Fig. 9b, c and Table S5). Tested against the cell-only control in a difference-in-differences framework, the treated changes were non-significant in every interval but one: *S. aureus* + Chem-AgNP showed *Δ*(4 − 1 h) = +0.67 mAU (*p* = 0.02), with a corresponding consecutive *Δ*(4 − 3 h) = +0.83 mAU.

**Fig. 9:**
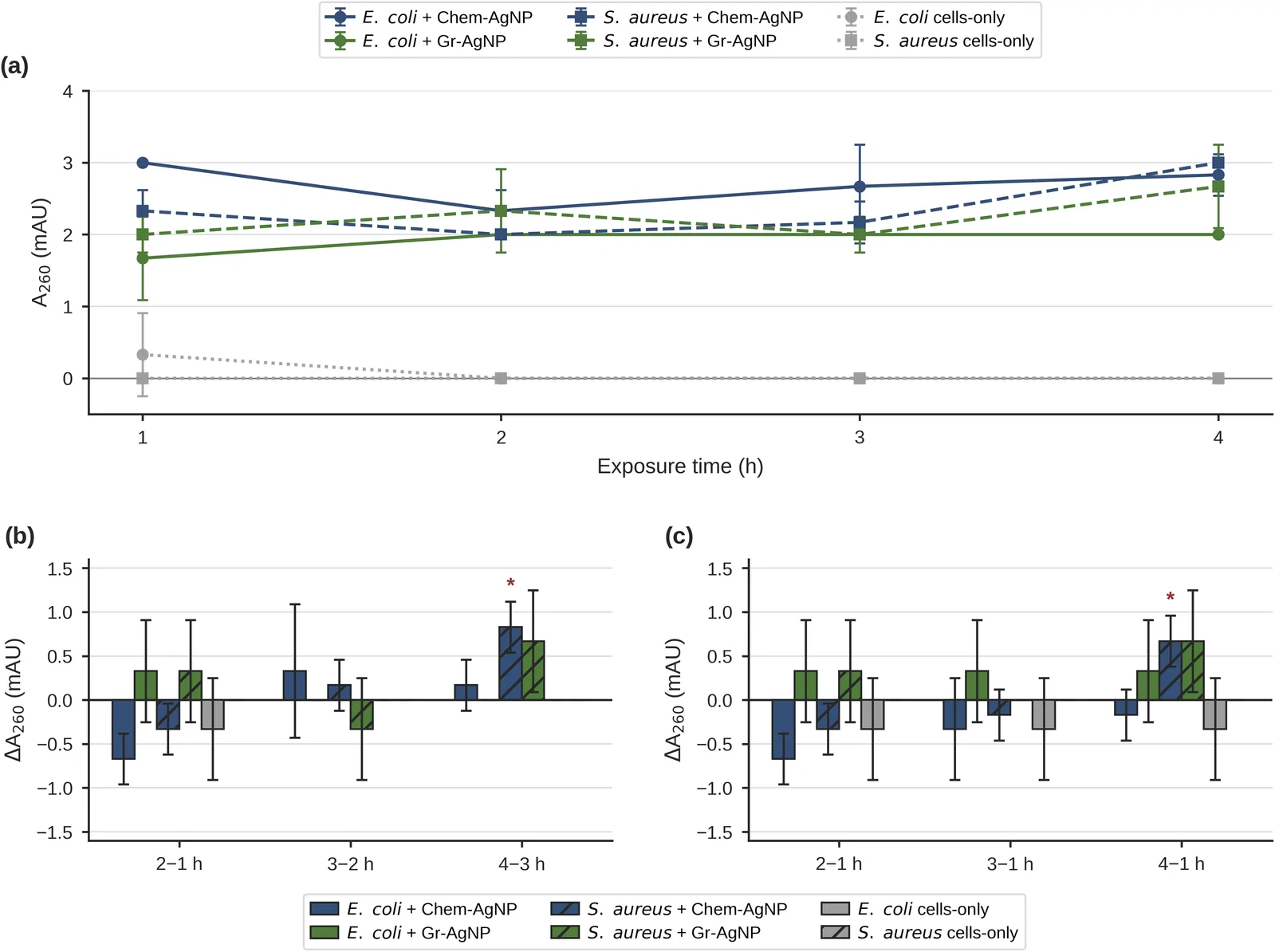
Time-resolved Δ*A*_260_ analysis of AgNP-treated *Escherichia coli* and *Staphylococcus aureus versus* cell-only control. (a) Mean supernatant *A*_260_ over 1–4 h: treated groups sit at 2–3 mAU while cell-only controls remain near 0 mAU. (b) Consecutive-interval Δ*A*_260_ and (c) Δ*A*_260_ relative to the 1 h reference; individual step changes are small and scattered around zero, with control deltas flat. Only *S. aureus* + Chem-AgNP at 4 − 1 h reaches significance (*, *p* < 0.05). Plain bars and solid lines denote *E. coli*; hatched bars and dashed lines denote *S. aureus*.

The two kinetic analyses operate at different resolutions. Increased extracellular *A*_260_ was already evident at the first 1 h measurement, whereas subsequent changes were modest. Linear regression detected a significant positive time trend in two of the four organism/particle combinations, while most individual interval-to-interval changes were not significant. The most conservative interpretation is therefore an early-onset release followed by limited additional accumulation over the 1–4 h window, rather than a uniformly progressive increase in every group.

### Three complementary methods support envelope permeabilisation

The core finding of this study is that sub-inhibitory AgNP exposure compromises bacterial-envelope integrity and is accompanied by loss of intracellular material. Three complementary readouts support this interpretation. Flow cytometry showed an enlarged Hoechst-low/negative subpopulation, SEM revealed collapsed and ghost-like envelopes, and the supernatant Δ*A*_260_ assay detected increased UV-absorbing material in the extracellular medium. No single one of these measurements is sufficient on its own: the cytometric signal reports reduced DNA-associated fluorescence rather than DNA release, the micrographs are qualitative, and the absorbance assay is a bulk measurement close to its detection floor. Their convergence is what supports the interpretation. For the chemical benchmark, the *A*_260_/*A*_280_ ratios were consistent with a predominantly nucleic-acid contribution; for Gr-AgNP, the lower ratios indicate that protein- or corona-derived material may also contribute. Accordingly, the combined data support envelope damage and intracellular-material release, while the chemical nature of the released material is less certain for Gr-AgNP. The assay panel was designed so that each technique addresses the limitations of the others; the rationale is summarised in Table S7.

The time-resolved analysis adds information that a single end-point measurement cannot provide. Release was already detectable at the first 1 h measurement and showed only modest subsequent increases. This pattern is consistent with early envelope damage followed by continued low-level efflux. However, because statistically significant time-dependent slopes were observed in only two of the four organism/particle combinations, and because regulated bacterial death pathways were not measured directly, the present data should not be interpreted as distinguishing physical disruption from specific regulated cell-death mechanisms.

### Structure–activity relationships and comparison with chemically reduced silver

The physicochemical data provide context for the biological responses but do not by themselves establish causality. Both preparations showed a dominant Ag (111) reflection, whereas they differed in phase composition, particle-size distribution, morphology and surface charge (Table 8). The white-tea preparation was more uniform and carried a biomolecular corona, while the chemical preparation contained an AgCl phase and a broader spherical/rod population. These differences may influence Ag^+^ availability and particle–cell interactions; however, Ag^+^ release and corona composition were not quantified directly in the present study.

**Table 8:** Head-to-head comparison of the white-tea green route (Gr-AgNP) and the chemical route (Chem-AgNP) across the properties that govern the antibacterial behaviour

Property	Gr-AgNP (white tea)	Chem-AgNP (citrate)	Advantage
Phase composition	Single-phase Ag^0^ + organic corona	Two-phase Ag + AgCl	Green
Crystallite size, (111)	15.6 nm (better ordered)	11.1 nm	—
Dislocation density	3.7 × 10^15^ m^−2^	7.0 × 10^15^ m^−2^	Green
SEM particle size/CV	28.3 nm/27%	31.9 nm/50%	Green
Morphology	Single population	Mixed spheres + rods (tail ≈ 140 nm)	Green
Zeta potential	−19.6 mV	−13.4 mV	Green
Polydispersity index	0.319	0.466	Green
MIC (µg mL^−1^)	4.7	38.5	Green
Capping/synergy	Bioactive polyphenol corona	Citrate only	Green
Hoechst-low/negative fraction (max)	3.0× (*E. coli*); 1.8× (*S. aureus*)	8.0× (*E. coli*); 1.4× (*S. aureus*)	Organism-dependent
Nucleic-acid leakage Δ*A*_260_	+1.8 to +2.3 mAU	+2.4 to +2.6 mAU	Comparable
*A*_260_/*A*_280_ of released material	1.17–1.35	2.11–2.17	Chemical
Reagents and waste	Renewable, solvent-free	Toxic reductant, AgCl by-product	Green

The cytometric responses were organism- and preparation-dependent, but the mechanistic exposures were not fully dose-matched. In particular, Gr-AgNP was tested at 1.19 µg mL^−1^ in *E. coli* and at 2.37 µg mL^−1^ in *S. aureus*—one-quarter and one-half of the respective MIC—which precludes a strict cross-species or head-to-head potency comparison. Baseline Hoechst-low/negative fractions also differed between the organisms, further affecting fold-change estimates: *E. coli* had a very low control fraction (≈1%) and hence a large relative response, whereas *S. aureus*, with its thick peptidoglycan wall, carried a high baseline (≈13%) that compresses the detectable dynamic range. The thicker Gram-positive wall is the standard explanation for reduced silver susceptibility.^4,7^ The cytometry results should therefore be interpreted as evidence of treatment-associated changes within each experimental condition rather than as a quantitative ranking of intrinsic membrane-disrupting potency.

Chemically prepared AgNPs provide a useful benchmark because their antibacterial effects have been associated with Ag^+^ release, oxidative stress, membrane damage and interactions with nucleic acids and thiol-containing proteins.^4,9,10,18^ Their activity depends strongly on synthesis conditions, particle properties and phase composition.^20,25,26,86^ In the present study, the chemical preparation therefore serves primarily as an experimentally matched comparator rather than as an idealised pure-metallic AgNP reference, because XRD showed that it contained both Ag and AgCl.

Our chemical benchmark produced the largest cytometric response observed in *E. coli* and the highest extracellular *A*_260_ values, but it was a less uniform, two-phase Ag + AgCl preparation. Its *A*_260_/*A*_280_ ratio also provided stronger evidence that the released material contained a substantial nucleic-acid fraction. The practical comparison is therefore not one-sided: the chemical preparation showed the larger cytometric effect in *E. coli* and analytically cleaner identification of the released material, whereas the white-tea preparation had a substantially lower MIC when normalised to total silver and showed more uniform colloidal characteristics. Because the Gr-AgNP exposures were unequal between the two organisms, these data should not be interpreted as a strict head-to-head potency comparison across all endpoints.

### Comparison with tea-mediated and other plant-mediated silver nanoparticles

A quantitative comparison with the recent literature is assembled in Table S6. Three features of that compilation govern how it should be read. First, particle size is reported by techniques that are not interchangeable: electron microscopy measures the dry metallic core, dynamic light scattering returns a hydrodynamic diameter that includes the capping corona and its solvation layer, and Scherrer analysis of X-ray line broadening yields a crystallite dimension. For a single preparation, these can differ several-fold—Dayana *et al.* report ≈10 nm by high-resolution transmission electron microscopy, 25.47 nm by X-ray diffraction and 45.79 nm by light scattering for the same material^36^^36^—so sizes are tabulated by technique rather than pooled. Second, antibacterial potency is reported on mutually incompatible dose scales: as a mass concentration of colloid, as a volume of undiluted suspension, as a particle number concentration, or as a mass of a composite. Only a mass concentration referenced to measured silver is directly comparable with the values reported here. Third, an MIC and an inhibition-zone diameter are different endpoints and cannot be interconverted. Cross-study rankings are therefore indicative rather than definitive, and the comparisons that follow are framed accordingly.

Within those limits, the white-tea particles reported here (28.3 ± 7.7 nm by SEM) lie in the middle of the tea-mediated range, which spans 14.9 ± 1.4 nm for black tea,^60^ 19.85 ± 3 nm for roasted green tea,^62^ 25–55 nm for green tea^59^ and 29.564 nm for a green-tea preparation directed at extended-spectrum β-lactamase producers,^61^ and they are substantially smaller than the only white-tea particles for which a comparable measurement exists (49–89 nm by SEM, 51–56 nm by TEM).^66^ The colloidal comparison is the more instructive one. The green-tea preparation of Juzer *et al.* is almost indistinguishable from ours in core size (29.564 *versus* 28.3 nm) and in surface charge (*ζ* = −20.1 *versus* −19.6 mV), yet reports a hydrodynamic diameter of 695.4 nm—an order of magnitude above the *Z*-average of 148.8 ± 3.1 nm measured here.^61^ Two preparations that electron microscopy would call equivalent therefore differ profoundly in dispersion state, which is the state in which particles actually encounter bacteria. Reported zeta potentials across the plant-mediated literature cluster narrowly, with −15.9 mV for lavender,^51^ −18.9 mV for *Convolvulus arvensis*,^35^ −20.1 mV for green tea,^61^ −21 mV for *Melaleuca alternifolia*^36^ and ≈−26 mV for *Carica papaya*,^39^ so the white-tea value of −19.6 ± 0.8 mV is unremarkable among them, and the −39.02 mV reported for an *Emblica officinalis* preparation is the exception rather than the norm.^87^

Comparison on the basis of MIC is limited by the small number of tea-mediated studies that determine one: of the seven preparations compiled in Table S6, only two report an MIC. Göl *et al.* obtained 6.25 µg mL^−1^ against *E. coli*, *S. aureus* and *B. subtilis*, and 12.5 µg mL^−1^ against methicillin-resistant *S. aureus*, for black-tea AgNPs by broth dilution, with minimum bactericidal concentrations of 6.25–25 µg mL^−1^.^60^ Juzer *et al.* obtained 3.125 µg mL^−1^ against both *E. coli* EC-13 and *E. coli* ATCC and 0.781–1.56 µg mL^−1^ against *Klebsiella pneumoniae* and *A. baumannii* for green-tea AgNPs, but tested no Gram-positive organism.^61^ The remaining tea studies quantify activity on other scales entirely: Tran Khac *et al.* report inhibition zones at particle number concentrations of 1.25–5 × 10^10^ particles per mL against *P. aeruginosa* and *S. aureus*;^59^ Makvandi *et al.* report inhibition zones for a silver/multi-walled-carbon-nanotube composite applied at 100 mg mL^−1^;^65^ and Chavez-Granados *et al.* incorporate AgNPs at a fixed 4 µg mL^−1^ into an alginate–gelatin hydrogel for bone regeneration without any antibacterial determination.^62^ Against this background, the white-tea MIC of 4.7 µg mL^−1^—the same endpoint against *E. coli* ATCC 25922 and *S. aureus* ATCC 29213—is lower than the black-tea value reported by Göl *et al.* by a factor of 1.3 and higher than the green-tea *E. coli* value reported by Juzer *et al.* by a factor of 1.5. The white-tea preparation is therefore comparable in potency to the best-characterised tea-mediated particles rather than markedly superior to them.

A more exacting comparison is available from plant-mediated preparations assayed against the same reference strains. Al-Audah *et al.* determined MICs for *C. arvensis* AgNPs of 12.5 µg mL^−1^ against *E. coli* ATCC 25922 and 25 µg mL^−1^ against *S. aureus* ATCC 29213—the identical strains used here—with corresponding MBCs of 25 and 50 µg mL^−1^.^35^ On those strains the white-tea particles are 2.7-fold more potent against *E. coli* and 5.3-fold more potent against *S. aureus*. Samanta *et al.* report 20 µg mL^−1^ against multidrug-resistant *E. coli* and 30 µg mL^−1^ against multidrug-resistant *S. aureus* for *C. papaya* AgNPs,^39^ and Sil *et al.* report 9.76–19.53 ppm against *E. coli* for *E. officinalis* AgNPs.^87^ The white-tea MIC value lies below all of these. Two qualifications attach to that ordering. The first concerns strain identity: the *Convolvulus* and papaya comparisons involve resistant clinical isolates in part, and MICs determined against resistant isolates are not interchangeable with those determined against susceptible reference strains. The second is more consequential: none of these studies records whether its dose axis was referenced to an independent determination of elemental silver.

This second qualification merits closer attention, since it identifies the weakest link in any cross-study comparison of nanoparticle potency. A colloidal AgNP suspension carries silver as metallic particles, as adsorbed and dissolved Ag^+^, and—where chloride is present—as sparingly soluble AgCl; it also carries capping biomolecules that contribute mass but no silver. A dose expressed as “µg mL^−1^ of AgNP” without an elemental determination is therefore a dose of suspension rather than of silver, and two such doses are comparable only if the suspensions happen to share both silver content and speciation. No study compiled in Table S6 reports an inductively coupled plasma or atomic-absorption determination of the silver content underlying its MIC. In the present work, each MIC was referenced to the ICP-MS-determined total silver of the corresponding stock (28.5 µg mL^−1^ for Gr-AgNP, 102.5 µg mL^−1^ for Chem-AgNP), so that the eight-fold mass-specific contrast between the two preparations is attributable to the silver rather than to the suspension. Wider adoption of this normalisation would materially improve comparability, since in its absence the apparent potency ranking across the green-synthesis literature partly reflects the concentration at which each colloid happened to be prepared.

Two further features of the present preparations are relevant to this comparison. First, XRD detected no crystalline silver-salt impurity in the white-tea product, whereas the chemical benchmark contained AgCl; phase composition is rarely reported in the green-synthesis literature, yet it determines what fraction of the nominal silver dose is available as soluble Ag^+^. Second, the biological evidence here rests on three convergent readouts—flow cytometry, electron microscopy and time-resolved supernatant absorbance—rather than on a single inhibition endpoint, and the *A*_320_ analysis showed that sample-processing differences can distort nanoparticle leakage measurements. Both observations argue for careful phase characterisation and for matched processing of controls whenever antibacterial nanomaterials are compared across studies.

### White tea and green tea as reducing and capping environments

The two grades differ in manufacture rather than in botanical origin, and the difference is consequential for synthesis. White tea is withered and dried with minimal leaf disruption; green tea is steamed or pan-fired to inactivate polyphenol oxidase before rolling; black tea is deliberately oxidised, converting monomeric catechins into theaflavins and thearubigins.^69^ Because the reducing capacity of a tea extract resides largely in its monomeric phenolics, this processing hierarchy predicts that black tea should be the weakest reductant of the three—a prediction relevant to the interpretation of Göl *et al.*, whose ceramic-bound preparation derives from black rather than green tea and is labelled accordingly in Table S6.^60^

It does not follow, however, that white tea is a richer catechin source than green tea. In the most directly comparable dataset available, in which white and green teas were extracted and analysed under identical ISO conditions, total polyphenols averaged 21.54 g per 100 g (range 16.23–25.95) in white tea against 19.18 g per 100 g (13.7–24.7) in green tea, and total catechins 13.22 g per 100 g (7.94–16.56) against 12.95 g per 100 g (9.89–17)—differences that are small relative to the within-grade ranges.^69^ The consistent differences lie in distribution rather than total: white tea averaged higher epigallocatechin gallate (EGCG, 8.00 *versus* 6.75 g per 100 g) and markedly higher caffeine (4.85 *versus* 2.90 g per 100 g), but lower epigallocatechin (1.11 *versus* 2.84 g per 100 g) and lower flavonol glycosides (0.61 *versus* 1.1 g per 100 g).^69^ Those authors reject the widely repeated claim that white tea is substantially higher in catechins than green tea, and report that the catechin-to-total-phenolics ratio does not discriminate between the two grades.^69^ Broader surveys agree that cultivar, plucking standard, season and extraction solvent dominate over grade, with white- and green-tea catechin ranges overlapping almost entirely.^70,71^ White tea is therefore better characterised as a compositionally distinct reducing and capping environment than as a uniformly richer one.

The mechanistic consequence of that distinction is a shift in the balance of galloylated to non-galloylated catechins rather than a change in total reducing capacity. Reduction of Ag^+^ by tea phenolics proceeds through electron donation from *ortho*-dihydroxy (catechol) and galloyl hydroxyls, which are oxidised to the corresponding quinones; the oxidised species then chelate the nascent particle surface through –OH and C=O groups and, where co-extracted proteins are present, through –NH functionalities.^30,33^ EGCG and epicatechin gallate carry the greatest number of such donor groups per molecule, so an extract weighted towards galloylated catechins—as white tea is relative to green tea in the dataset above—supplies both more rapid reduction and a denser capping layer. The infrared data reported here are consistent with this chemistry: the C=O/amide I and C–O bands show the largest shifts on particle formation (1605 → 1636 cm^−1^ and 1040 → 1001 cm^−1^; Fig. 5 and Table S1), identifying carbonyl and C–O functionalities as those most directly engaged with the silver surface. A corona of this kind accounts for the properties measured here in a self-consistent manner. It restricts growth, giving the single-mode size distribution resolved by SEM; it contributes the negative surface charge measured by electrophoretic light scattering; and it adds a steric component to stabilisation alongside the electrostatic one, which is the standard explanation for colloids that remain stable at zeta potentials inside the conventional |*ζ*| < 30 mV instability window.^51^ It also offers a plausible account of why no crystalline AgCl was detected in the biogenic product, since a dense phenolic corona both excludes chloride from the growing surface and sustains a locally reducing environment.

Two limitations bound this interpretation. First, the white-tea extract used here was not itself assayed for catechin or total-polyphenol content, so the compositional argument rests on the literature rather than on a determination performed on the present feedstock. Second, no study has yet synthesised AgNPs from white and green tea under otherwise identical conditions, and such a design is the only one capable of isolating a grade effect from the many other variables that differ between reports. A controlled comparison of this kind, in which tea grade is varied while extraction, reduction and assay conditions are held constant, would establish whether processing exerts a reproducible influence on particle size, dispersion state and silver-normalised potency, and represents the natural extension of the present work.

### Significance, limitations and outlook

This study contributes a physicochemically characterised white-tea AgNP preparation, an experimentally matched chemical benchmark and complementary measurements of bacterial-envelope damage. It also identifies a sample-processing mismatch that can bias *A*_260_ leakage calculations and shows the practical value of checking residual particle-associated absorbance and scattering. The white-tea synthesis avoids conventional toxic reducing agents; however, no quantitative life-cycle or environmental assessment was performed, so broader sustainability claims are not warranted from the present data alone.

Several limitations bear on the interpretation of these results. Replicates were technical rather than independent biological replicates (*n* = 3), which limits statistical inference. Although MIC values were determined separately for each organism, the Gr-AgNP exposures were unequal between species (1.19 µg mL^−1^ for *E. coli*, 2.37 µg mL^−1^ for *S. aureus*), so the cytometric and leakage data do not support a cross-species ranking of intrinsic potency. The low Gr-AgNP *A*_260_/*A*_280_ ratios (1.17–1.35) may reflect protein or corona-derived contributions. Finally, mammalian cytotoxicity and haemocompatibility were not assessed, so no therapeutic window is implied.

## Conclusions

White-tea-mediated synthesis yielded a uniform, single-phase metallic silver nanoparticle preparation with no detectable crystalline silver-salt impurity, and one that reached its minimum inhibitory concentration at approximately one-eighth of the silver mass required by the chemically prepared benchmark assayed alongside it. For each preparation, the MIC was determined first, and the sub-inhibitory concentrations used in all subsequent mechanistic work were then set relative to that value, so that every downstream measurement is referenced to an independently established potency scale.

Three independent readouts—flow cytometry, field-emission electron microscopy and time-resolved supernatant absorbance—converged on AgNP-associated damage to the bacterial envelope and on release of intracellular material. Their agreement is the substance of the evidence: each readout offsets a specific weakness in the others, and no single measurement is asked to carry the interpretation alone.

The *A*_320_ analysis produced a result of wider applicability than the material itself. Mismatched processing of nanoparticle-treated and particle-only controls inflates apparent *A*_260_ leakage, and the scatter correction described here removes that artefact. The procedure requires no additional instrumentation and can be adopted directly by any laboratory reporting nucleic-acid leakage from nanoparticle-treated cells.

Extracellular release was already detectable at the first hourly measurement, with more modest and less uniform increases thereafter. The data therefore establish early envelope damage, while leaving open the distinction between direct physical disruption and downstream cell-death pathways—a question that the single-cell resolution demonstrated here now makes directly addressable.

Taken together, these results establish white tea as a productive phytochemical medium for preparing well-defined antibacterial silver nanoparticles, and they illustrate what becomes visible once phase composition is resolved and dose is referenced to measured silver rather than to colloid volume. Both practices are straightforward to adopt and would materially improve comparability across the green-synthesis literature. The next steps follow directly from the design used here: matched sub-inhibitory exposures across species, independent biological replication, DNA-specific quantification of the released material, and extension to a broader panel of pathogens, including multidrug-resistant clinical isolates, alongside mammalian cytotoxicity and haemocompatibility assessment. The preparation characterised in this work provides a well-defined starting point for that programme.

## Author contributions

Mohammed O. H. Jaber: conceptualisation, investigation, methodology, formal analysis, visualisation, writing – original draft. Mehmet Yılmaz: validation, resources, funding acquisition, writing – review and editing. Aslı Yılmaz: supervision, methodology, validation, resources, funding acquisition, writing – review and editing. All authors read and approved the submitted version.

## Conflicts of interest

There are no conflicts to declare.

## Supplementary Material

RA-OLF-D6RA08462A-s001

## Data Availability

The data supporting this article have been included as part of the supplementary information (SI). Supplementary information: the full synthesis protocol (Section S1 and Table S8), the flow-cytometry acquisition and gating detail (Section S2), the diagnostic infrared band assignments (Table S1), the replicate-level flow-cytometry data (Table S2), the complete measured absorbances underlying the leakage and kinetic analyses (Tables S3–S5), the comparison with representative green-synthesised silver nanoparticles (Table S6), the assay-panel rationale (Table S7) and the morphology-resolved particle-size distributions (Fig. S1). Together, these contain every numerical value from which the figures and tables of this article were generated. Additional raw datasets generated and analysed during the present study, including unprocessed X-ray diffractograms, SEM image sets and particle-measurement tables, light-scattering correlograms, FTIR spectra, flow-cytometry FCS files and the complete absorbance readings underlying the leakage and kinetic analyses, are available from the corresponding authors on reasonable request. References cited in the SI are listed in the reference list of this article. See DOI: https://doi.org/10.1039/d6ra08462a.
