# Supplementary material for: Single-phase silver nanoparticles from white tea: colloidal quality, mass-specific potency and bacterial envelope permeabilisation
Source: RSC Adv. 2026 Sep 28;16(45):57931–49. doi: 10.1039/d6ra08462a (PMC13618064; doi:10.1039/d6ra08462a)
Supplement: Supplementary file 1 [file RA-016-D6RA08462A-s001.pdf]

Supplementary Information (SI) for RSC Advances.
This journal is © The Royal Society of Chemistry

RSC ADVANCES | SUPPLEMENTARY INFORMATION

Single-phase silver nanoparticles from white tea: colloidal quality, mass-specific potency and bacterial envelope permeabilisation

Mohammed O. H. Jaber[a,b], **Mehmet Yılmaz**[c,d] and **Aslı Yılmaz**[a,e]

[a] Department of Molecular Biology and Genetics, Atatürk University, 25240 Erzurum, Türkiye

[b] Department of Medical Sciences, University College of Science and Technology (UCST), Gizan Al-Najjar, P.O. Box 8, Khan Younis, Gaza Strip, Palestine

[c] Department of Chemical Engineering, Atatürk University, 25240 Erzurum, Türkiye

[d] Department of Nanoscience and Nanoengineering, Atatürk University, 25240 Erzurum, Türkiye

[e] Institute for Drugs, Vaccines and Biotechnology, Atatürk University, 25240 Erzurum, Türkiye

Corresponding authors: mohammedoh.jaber20@ogr.atauni.edu.tr; asli.yilmaz@atauni.edu.tr

Contents

Section S1 Detailed synthesis protocol

Section S2 Flow-cytometry acquisition, gating and controls

Fig. S1 Morphology-resolved size distributions for Chem-AgNP

Table S1 Diagnostic infrared band shifts on green synthesis

Table S2 Replicate-level flow-cytometry data

Table S3 Measured absorbance of all sample sets (A_{320}, A_{260}, A_{280})

Table S4 Mean supernatant A_{260} at each exposure timepoint

Table S5 ΔA_{260} between timepoints

Table S6 MIC, particle size and dose basis for tea-mediated and other plant-mediated AgNPs

Table S7 Assay panel and rationale for each component

Table S8 Synthesis parameters for Gr-AgNP and Chem-AgNP

Section S1 Detailed synthesis protocol

This section reports the complete preparative procedure for both silver nanoparticle preparations, so that either can be reproduced without recourse to secondary sources. All glassware was cleaned before use and all solutions were prepared in distilled water unless otherwise stated. Both syntheses were carried out with protection from ambient light in order to prevent photoreduction of Ag^+, and reaction progress was monitored in both cases by the characteristic colour change from pale yellow to dark brown and by the appearance of the surface-plasmon-resonance (SPR) absorption band at 400–430 nm. The parameters are collected in Table S8.

S1.1 White-tea extract preparation

Dried white-tea (*Camellia sinensis*) leaves (2 g) were added to 100 mL of distilled water, giving a 2% (w/v) leaf-to-water ratio. The mixture was heated to 80–90 °C and simmered for 15–20 min, then allowed to cool to room temperature. The cooled infusion was clarified first through qualitative filter paper and then through a 0.22 μm syringe filter. The clarified extract was stored at 4 °C until use.

S1.2 Green (biogenic) synthesis of Gr-AgNP

An aqueous silver nitrate solution (0.1 mM; 0.0017 g $AgNO_3$ in 100 mL distilled water) was prepared fresh. The white-tea extract and the $AgNO_3$ solution were combined in a 1 : 1 (v/v) ratio — for example, 1 mL of extract with 1 mL of 0.1 mM $AgNO_3$. The pH of the reaction mixture was adjusted to 8.0–9.0 with 0.1 M NaOH. The mixture was then heated at 45–60 °C under constant magnetic stirring for 2–4 h, with protection from light throughout. Formation of Gr-AgNP was followed by the colour change from pale yellow to dark brown and confirmed by the SPR band at 400–430 nm; the reaction was judged complete when no further deepening of the colour or growth of the SPR band was observed.

S1.3 Chemical (citrate) synthesis of Chem-AgNP

An aqueous silver nitrate solution (0.1 mM; 0.0017 g $AgNO_3$ in 100 mL distilled water) was prepared fresh, and a 1.5% (w/v) solution of anhydrous trisodium citrate was prepared separately (0.075 g in 5 mL distilled water; 58.1 mM). The pH of the $AgNO_3$ solution was adjusted to 8.0–9.0 with 0.1 M NaOH. The $AgNO_3$ solution (100 mL) was heated to 90 °C, and 0.588 mL of the trisodium citrate solution was then added under constant magnetic stirring; stirring was continued for 15–30 min at that temperature, with protection from light throughout. This corresponds to 8.82 mg (3.42×10^{-5} mol) of anhydrous trisodium citrate ($M = 258.07$ g mol^{-1}) added to 1.00×10^{-5} mol of Ag^+, that is, a citrate-to-silver molar ratio of approximately 3.4 : 1 and a citrate concentration of 0.34 mM in the reacting mixture. Reduction was followed by the same colour change from pale yellow to dark brown and confirmed by the SPR band at 400–430 nm. The route was operated with the intention of producing particles below 20 nm.

Both routes were prepared from the same 0.1 mM silver nitrate stock. In the citrate route that stock forms the reaction medium directly; in the biogenic route it is combined 1 : 1 (v/v) with the extract, so that the silver concentration in the reacting mixture is 0.05 mM. The two preparations therefore differ in their reducing and capping chemistry rather than in the silver precursor offered to it. They are further matched by having been produced and assayed in parallel and, critically, by dose normalisation: every antibacterial and mechanistic exposure reported in this work is set against the *measured* total silver content of the purified stock, determined by ICP-MS, rather than against a nominal precursor concentration. Section S1.4 gives those measured values.

S1.4 Purification and storage

Both preparations were purified identically. The reaction suspension was centrifuged at 12 000 rpm for 30 min to collect the nanoparticles, and the pellet was washed three times with distilled water (ethanol may be substituted), with redispersion between cycles, in order to remove unreacted precursor, excess reductant and loosely associated organics. The washed pellet was finally resuspended in ultrapure (Milli-Q) water and stored protected from light until use; the concentration of each purified stock is set by the volume of water used at this step, and was measured directly rather than inferred from the precursor concentration. Purified stocks were redispersed immediately before every measurement. The total silver content of each purified stock was determined by inductively coupled plasma–mass spectrometry (ICP-MS), giving approximately 28.5 μg mL^{-1} for Gr-AgNP and 102.5 μg mL^{-1} for Chem-AgNP.

Section S2 Flow-cytometry acquisition, gating and controls

Envelope-associated loss of DNA staining was assessed at the single-cell level after a 20 h exposure using Hoechst 33342, a membrane-permeant minor-groove double-stranded DNA stain that enters cells irrespective of viability. Fluorescence was acquired in the PB450-A (Pacific Blue) channel and 10 000 events were recorded for every sample.

Gating strategy. Events were first gated on forward scatter (FSC) *versus* side scatter (SSC) to exclude instrument noise and sub-bacterial debris and to define the bacterial population. A single scatter gate was drawn for each organism and applied unchanged to every sample within a run, so that treated and control samples were compared on an identical population definition. Within that scatter gate, the dye-negative/dye-positive boundary in the PB450-A channel was set from the no-dye (unstained) control and applied uniformly across all samples in the run. All percentages quoted in the main text and in Table S2 are therefore percentages of the scatter-gated population.

Controls acquired. Each run included, for each organism, (i) an unstained, untreated control, used to place the dye-negative/positive boundary; and (ii) a stained, untreated control, used as the reference against which fold-changes are expressed. The stained, untreated controls consistently displayed the expected DNA-associated fluorescence, confirming that the staining procedure was capable of detecting intracellular DNA in these bacterial populations under the conditions used.

Controls not acquired, and the consequences for interpretation. Nanoparticle-only fluorescence and scatter controls were not acquired. A residual particle-associated contribution to the scatter-gated population therefore cannot be formally excluded, and this is stated among the limitations of the main text. Accordingly, a Hoechst-low/negative event is interpreted throughout as a reduction in detectable DNA-associated fluorescence and not as direct evidence of DNA release. The cytometric data are used as one of three convergent readouts and are interpreted together with the supernatant ΔA_{260} measurements, which quantify nucleic-acid-associated material in the extracellular medium, and with field-emission scanning electron microscopy, which provides morphological evidence of envelope alteration.

Replication. Experiments were performed three times on separate days using two independent synthesis batches (n = 3). Replicate-level values are given in Table S2.

Supplementary figure

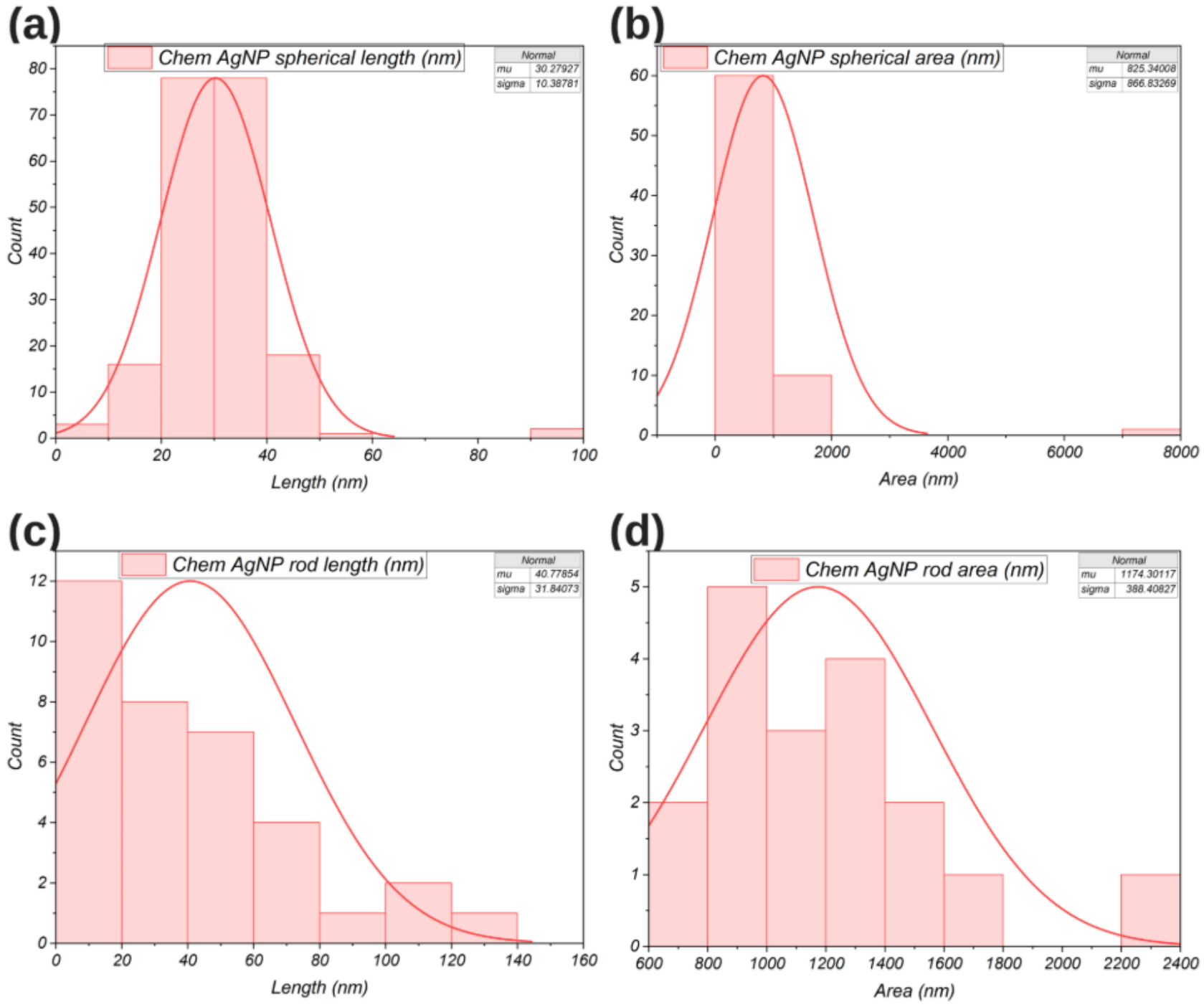

Fig. S1 Morphology-resolved scanning electron microscopy size distributions for the chemically reduced sample (Chem-AgNP), with Gaussian fits. (a and b) Length and projected area of the spherical sub-population (≈ 85% by number; *μ* = 30.3 nm, *σ* = 10.4 nm). (c and d) Length and projected area of the rod sub-population (≈ 15%; *μ* = 40.8 nm, *σ* = 31.8 nm). The rods are longer and far more disperse than the spheres and are the origin of the right-skewed tail seen in Fig. 2c of the main text. Even the spherical sub-population alone (CV ≈ 34%) is broader than the entire Gr-AgNP population (CV ≈ 27%). These data are summarised in Table 3 of the main text.

Supplementary tables

Table S1 Diagnostic infrared band shifts on green synthesis (raw white-tea extract → Gr-AgNP). The two largest shifts identify the carbonyl and C–O functionalities most directly involved in Ag^+ reduction and subsequent capping

Functional group	Raw white tea (cm^{-1})	Gr-AgNP (cm^{-1})	Shift (cm^{-1})
O–H/N–H stretch (broad)	3300	3321	+21
C=O/amide I (protein carbonyl)	1605	1636	+31 **(key)**
C–O/C–N (alcohol, phenol, sugar)	1040	1001	−39 **(key)**
Lower fingerprint (Ag–O/framework)	610	615	+5

Table S2 Hoechst-low/negative (PB450-A) events as a percentage of the scatter-gated population, by treatment and replicate (n = 3). "Fold" is the mean relative to the corresponding stained control. Exposures are absolute concentrations of total silver (Table 5 of the main text). These are the replicate-level values summarised in Table 6 of the main text. A Hoechst-low/negative event denotes a reduction in detectable DNA-associated fluorescence and is not, on its own, direct evidence of DNA release (Section S2)

Sample/treatment	Rep 1	Rep 2	Rep 3	Mean ± SD	Fold
E. coli — stained control	1.22	1.36	1.03	1.20 ± 0.17	1.0×
E. coli — Chem-AgNP, 19.2 μg mL^{-1}	9.54	10.13	9.16	9.61 ± 0.49	8.0×
E. coli — Gr-AgNP, 1.19 μg mL^{-1}	3.08	3.95	3.64	3.56 ± 0.44	3.0×
S. aureus — stained control	13.74	12.95	12.15	12.95 ± 0.80	1.0×
S. aureus — Chem-AgNP, 19.2 μg mL^{-1}	17.04	17.64	19.51	18.06 ± 1.29	1.4×
S. aureus — Gr-AgNP, 2.37 μg mL^{-1}	22.68	21.90	24.40	22.99 ± 1.28	1.8×

Table S3 Measured absorbance of every sample set across the 1–4 h window (mean ± SD, n = 3). Treated A_{320} lies at or below the cells-only level and far below the unprocessed AgNP-only control, confirming that filtration removed essentially all residual nanoparticles and that the cells-only set is the correct background for the leakage calculation

Sample set	A_{320}	A_{260}	A_{280}
E. coli cells-only	0.0566 ± 0.0010	0.0001 ± 0.0003	0.0000 ± 0.0000
E. coli + Chem-AgNP	0.0523 ± 0.0005	0.0027 ± 0.0004	0.0013 ± 0.0003
E. coli + Gr-AgNP	0.0531 ± 0.0010	0.0019 ± 0.0003	0.0014 ± 0.0005
S. aureus cells-only	0.0578 ± 0.0021	0.0000 ± 0.0000	0.0000 ± 0.0000
S. aureus + Chem-AgNP	0.0523 ± 0.0002	0.0024 ± 0.0004	0.0011 ± 0.0004
S. aureus + Gr-AgNP	0.0540 ± 0.0022	0.0023 ± 0.0005	0.0019 ± 0.0010
AgNP-only, chemical (unprocessed)	0.0808 ± 0.0000	0.0022 ± 0.0000	0.0015 ± 0.0000
AgNP-only, green (unprocessed)	0.0828 ± 0.0000	0.0033 ± 0.0000	0.0018 ± 0.0000

Table S4 Mean supernatant A_{260} (mAU, n = 3) at each exposure timepoint. Treated groups sit at 2–3 mAU throughout; cells-only controls remain at or near zero

Group	1 h	2 h	3 h	4 h
E. coli + Chem-AgNP	3.00	2.33	2.67	2.83
E. coli + Gr-AgNP	1.67	2.00	2.00	2.00
E. coli cells-only	0.33	0.00	0.00	0.00
S. aureus + Chem-AgNP	2.33	2.00	2.17	3.00
S. aureus + Gr-AgNP	2.00	2.33	2.00	2.67
S. aureus cells-only	0.00	0.00	0.00	0.00

Table S5 ΔA_{260} between timepoints (mAU, mean ± SD, $n = 3$), given both for consecutive intervals and relative to the 1 h reference. *, $p < 0.05$ *versus* zero. Only *S. aureus* + Chem-AgNP reaches significance, and only in the intervals involving the 4 h reading

Group	Δ(2–1 h)	Δ(3–2 h)	Δ(4–3 h)	Δ(3–1 h)	Δ(4–1 h)
E. coli + Chem-AgNP	−0.67 ± 0.29	+0.33 ± 0.76	+0.17 ± 0.29	−0.33 ± 0.58	−0.17 ± 0.29
E. coli + Gr-AgNP	+0.33 ± 0.58	+0.00 ± 0.00	+0.00 ± 0.00	+0.33 ± 0.58	+0.33 ± 0.58
E. coli cells-only	−0.33 ± 0.58	+0.00 ± 0.00	+0.00 ± 0.00	−0.33 ± 0.58	−0.33 ± 0.58
S. aureus + Chem-AgNP	−0.33 ± 0.29	+0.17 ± 0.29	+0.83 ± 0.29 *	−0.17 ± 0.29	+0.67 ± 0.29 *
S. aureus + Gr-AgNP	+0.33 ± 0.58	−0.33 ± 0.58	+0.67 ± 0.58	+0.00 ± 0.00	+0.67 ± 0.58
S. aureus cells-only	+0.00 ± 0.00	+0.00 ± 0.00	+0.00 ± 0.00	+0.00 ± 0.00	+0.00 ± 0.00

Table S6 Quantitative comparison of the white-tea particles reported here with representative tea-mediated and other plant-mediated silver nanoparticles. Particle sizes are listed separately by measurement technique, because electron-microscopic core diameters, dynamic-light-scattering hydrodynamic diameters and Scherrer crystallite sizes are not interchangeable and may differ several-fold for the same preparation. MIC values are given only where the source study determined one; where a different endpoint was used (inhibition-zone diameter) or where the dose cannot be converted to a mass concentration of silver, this is stated rather than estimated. The penultimate column records whether the dose axis was referenced to an independent elemental determination of silver by inductively coupled plasma mass spectrometry or atomic absorption; only the present work reports such a determination, and this is the principal limitation on cross-study potency comparison. Parameters not reported in a source study are marked accordingly. Reference numbers correspond to the main-text reference list.

Source / feedstock (tea grade)	Core size / nm (technique)	Hydrodynamic size / nm; PdI	□ / mV	MIC / μg mL^{-1} (*E. coli* / *S. aureus*)	Other reported MIC or activity endpoint	Ag-normalised dose	Ref.
White tea, *C. sinensis* (Gr-AgNP, this study)	28.3 ± 7.7 (SEM)	148.8 ± 3.1; 0.319	−19.6 ± 0.8	4.7 / 4.7	ΔA_{260} +1.8 (*E. coli*) and +2.3 (*S. aureus*) mAU; Hoechst-low/negative fraction 3.0× and 1.8×	Yes (ICP-MS)	—
Citrate reduction (Chem-AgNP, this study)	Bimodal (SEM)	72.9 ± 0.3; 0.466	−13.4 ± 0.7	38.5 / 38.5	ΔA_{260} +2.4 to +2.6 mAU; contains a crystalline AgCl phase	Yes (ICP-MS)	—
White tea leaf	51–56 (TEM); 49–89 (SEM)	Not reported	Not reported	Not tested	MIC 0.625 (*A. baumannii*, *P. aeruginosa*), 2.5 (*B. subtilis*), 10 (*S. haemolyticus*, *C. albicans*); antibiofilm inhibition 5–92%	No	66
White tea (endodontic irrigant)	30–35 (TEM)	Not reported	Not reported	Not tested	No MIC determined; inhibition zone 32 ± 2 mm against *E. faecalis* at 50 μL of colloid	No (μL colloid)	67
White tea / montmorillonite composite	19.77 ± 3.82; 15.87 ± 2.38 (TEM); ≈16 (XRD)	Not reported	Not reported	Not tested	No antibacterial testing performed; antioxidant (DPPH) and MOLT-4 cytotoxicity only	n/a	68

Source / feedstock (tea grade)	Core size / nm (technique)	Hydrodynamic size / nm; PdI	□ / mV	MIC / μg mL⁻¹ (*E. coli* / *S. aureus*)	Other reported MIC or activity endpoint	Ag-normalised dose	Ref.
Green tea leaf	25–55 (TEM)	Not reported	Not reported	Not tested	No MIC; inhibition zones against *P. aeruginosa* and *S. aureus* exceeding ampicillin; 96% methylene-blue degradation	No (particles mL^{-1})	59
Black tea, ceramic-bound	14.9 ± 1.4 (TEM)	Not reported	Not reported	6.25 / 6.25	MIC 6.25 (*B. subtilis*), 12.5 (MRSA); MBC 6.25 (*S. aureus*) to 25 (MRSA)	No	60
Green tea (ESBL-directed)	29.564 (HRTEM)	695.4 (988.8 overall)	−20.1	3.125 / not tested	MIC 0.781 (*K. pneumoniae* KP 01, *A. baumannii* SN-15), 1.56 (KP ATCC, AB MTCC 1926); MBC 25 (*E. coli*)	No	61
Roasted green tea (hydrogel)	19.85 ± 3 (TEM); 15–20 (XRD)	Not reported	Not reported	Not tested	No antibacterial determination; AgNP incorporated at a fixed 4 μg mL^{-1} into an alginate–gelatin hydrogel for bone regeneration	n/a	62
Oolong tea	25 (TEM); 31 (XRD)	78; 0.272	−34	Not tested	No MIC; applied at 10 μg mL^{-1} as a fruit-preservation coating	n/a	64
Green tea + multi-walled carbon nanotubes	Not stated numerically (TEM)	Not reported	Not reported	Inhibition zone only	No MIC; disc diffusion against *E. coli*, *Klebsiella*, *S. aureus* and *E. faecalis* at 100 mg mL^{-1} of composite	No	65
Emblica officinalis (phyto)	46.5	Not reported	−39.02	9.76–19.53 ppm / not tested	RNA-seq: arginine biosynthesis upregulated, flagellar genes (*flhDC*, *fliC*) downregulated	No	87
Carica papaya (phyto)	≈ 41	Not reported	≈ −26	20 / 30	MDR isolates; MBC and DAD assays concordant with MIC; ROS generation and DNA damage; haemocompatible to 50 μg mL^{-1}	No	39
Melaleuca alternifolia (phyto)	≈ 10 (HRTEM); 25.47 (XRD)	45.79; 0.335	−21	Not tested	No MIC; inhibition zones 7.3 mm (*P. aeruginosa*) and 7.0 mm (*S. pyogenes*) at 10 μL mL^{-1}	No	36
Convolvulus arvensis (phyto)	102.34–210.82 (SEM)	63; 0.267	−18.9	12.5 / 25	Same reference strains as this study (ATCC 25922, ATCC 29213); MBC 25 / 50; MIC 12.5 against *E. coli* ESBL and MRSA	No	35
Lavandula angustifolia (phyto)	16.2 ± 7.3 (TEM)	78.81; 0.331	−15.9	Inhibition zone only	No MIC; zones 9.56 mm (*E. coli*), 9.68 mm (*S. haemolyticus*), 7.99 mm (*P. aeruginosa*)	No	51

Table S7 Assay panel used in this study, with the rationale for each component and the specific weakness that other assays in the panel are intended to offset

Assay	What it measures	Why it was chosen	Key limitation covered by others
XRD (Scherrer, W–H)	Crystal phase, crystallite size, microstrain, dislocation density	Establishes phase purity and detects the AgCl secondary phase; the only method that distinguishes Ag^0 from silver salts	Bulk average; no morphology
SEM (particle)	Primary particle size, shape, distribution	Direct morphological measurement; resolves the mixed sphere/rod population	Small sampled population; dry state
DLS/ELS	Hydrodynamic size, PdI, zeta potential, conductivity	Reports behaviour in suspension — the state in which particles meet bacteria	Intensity-weighted; sensitive to aggregates
FTIR	Functional groups on the particle surface	Identifies which biomolecules reduce and cap the silver	Qualitative; no quantitation
ICP-MS	Total silver concentration	Normalises dose between the two preparations	No speciation between Ag^0 and Ag^+
Broth microdilution MIC (with MTT and OD_{600})	Growth-inhibitory concentration	Fixes the potency scale so that mechanistic work can be run at defined sub-MIC doses; determined independently for each organism	End-point only; no mechanism

Assay	What it measures	Why it was chosen	Key limitation covered by others
Flow cytometry (Hoechst 33342)	DNA-associated fluorescence per cell; Hoechst-low/negative fraction	Single-cell resolution; quantifies the proportion of the population affected	Reports reduced DNA staining rather than DNA release; no nanoparticle-only control; addressed by SEM and A_{260}
Field-emission SEM (cells)	Envelope ultrastructure after treatment	Visual confirmation that Hoechst-low/negative events correspond to physically emptied cells	Qualitative; no molecular identity; addressed by A_{260}/A_{280}
Supernatant A_{260} (with A_{280}, A_{320})	Released nucleic acid; protein contribution; residual particle load	Direct biochemical detection of the released material; A_{320} corrects the particle artefact	Bulk measure; near detection floor
Time-resolved ΔA_{260} (1–4 h)	Kinetics of release: regression slope and paired deltas	Distinguishes an instantaneous burst from progressive efflux	Limited power for sub-mAU steps (n = 3, technical)

Table S8 Synthesis parameters for the two silver nanoparticle preparations, as described in Section S1. Both routes were operated with protection from ambient light, and completion was judged from the colour change and the surface-plasmon-resonance band at 400–430 nm

Parameter	Gr-AgNP (white tea)	Chem-AgNP (citrate)
Reducing/capping agent	Aqueous white-tea (*C. sinensis*) leaf extract	Trisodium citrate, anhydrous (M = 258.07 g mol^{-1}), 1.5% (w/v) (0.075 g in 5 mL; 58.1 mM)
Plant material	2 g dried white-tea leaves per 100 mL distilled water (2%, w/v)	Not applicable
Extraction temperature and time	80–90 °C, 15–20 min, then cooled to room temperature	Not applicable
Filtration of extract	Qualitative filter paper, then 0.22 μm syringe filter	Not applicable
Extract storage before use	4 °C until use	Not applicable
$AgNO_3$ concentration	0.1 mM (0.0017 g in 100 mL distilled water)	0.1 mM (0.0017 g in 100 mL distilled water)
Reductant : $AgNO_3$ ratio	1 : 1 (v/v) extract : 0.1 mM $AgNO_3$ ([Ag] in the reacting mixture, 0.05 mM)	0.588 mL of 1.5% (w/v) trisodium citrate added to 100 mL of 0.1 mM $AgNO_3$ (8.82 mg citrate; citrate : Ag ≈ 3.4 : 1, mol/mol)
Reaction pH	8.0–9.0 (adjusted with 0.1 M NaOH)	8.0–9.0 (adjusted with 0.1 M NaOH)
Reaction temperature	45–60 °C	90 °C
Reaction time	2–4 h	15–30 min
Stirring	Constant magnetic stirring throughout	Constant magnetic stirring throughout
Light protection	Yes, throughout	Yes, throughout
Completion criterion	Pale yellow → dark brown; SPR band at 400–430 nm stable	Pale yellow → dark brown; SPR band at 400–430 nm stable
Purification	Centrifugation at 12 000 rpm, 30 min; pellet washed 3 × with distilled water	Centrifugation at 12 000 rpm, 30 min; pellet washed 3 × with distilled water
Storage of purified stock	Resuspended in ultrapure (Milli-Q) water; stored protected from light	Resuspended in ultrapure (Milli-Q) water; stored protected from light
Total Ag in purified stock (ICP-MS)	≈ 28.5 μg mL^{-1}	≈ 102.5 μg mL^{-1}
Target particle size	Not size-targeted; determined by the extract	< 20 nm

Abbreviations

AgNP, silver nanoparticle; AMR, antimicrobial resistance; Chem-AgNP, chemically reduced silver nanoparticle; CLSI, Clinical and Laboratory Standards Institute; CV, coefficient of variation; DLS, dynamic light scattering; DLVO, Derjaguin–Landau–Verwey–Overbeek; ELS, electrophoretic light scattering; ESBL, extended-spectrum β-lactamase; FCC, face-centred cubic; FSC, forward scatter; FTIR, Fourier-transform infrared spectroscopy; Gr-AgNP, green (white-tea) silver nanoparticle; ICP-MS, inductively coupled plasma–mass spectrometry; MDR, multidrug-resistant; MIC, minimum inhibitory concentration; MTT, 3-(4,5-dimethylthiazol-2-yl)-2,5-diphenyltetrazolium bromide; MWCNT, multi-walled carbon nanotube; PBS, phosphate-buffered saline; PdI, polydispersity index; ROS, reactive oxygen species; SEM, scanning electron microscopy; SPR, surface plasmon resonance; SSC, side scatter; UDEDM, uniform deformation energy density model; UDM, uniform deformation model; USDM, uniform stress deformation model; W–H, Williamson–Hall; XRD, X-ray diffraction.
